# Size-selective Pt siderophores based on redox active azo-aromatic ligands†

**DOI:** 10.1039/d0sc02683b

**Published:** 2020-08-20

**Authors:** Debabrata Sengupta, Sreetosh Goswami, Rajdeep Banerjee, Matthew J. Guberman-Pfeffer, Abhijeet Patra, Anirban Dutta, Rajib Pramanick, Shobhana Narasimhan, Narayan Pradhan, Victor Batista, T. Venkatesan, Sreebrata Goswami

**Affiliations:** School of Chemical Sciences, Indian Association for the Cultivation of Science Jadavpur Kolkata 700032 India icsg@iacs.res.in; NUSNNI-NanoCore, National University of Singapore Singapore 117411 Singapore sreetosh@u.nus.edu; NUS Graduate School for Integrative Sciences and Engineering (NGS), National University of Singapore Singapore 117456 Singapore; Department of Physics, National University of Singapore Singapore 117542 Singapore; Theoretical Sciences Unit, School of Advanced Materials, Jawaharlal Nehru Centre for Advanced Scientific Research Jakkur Bangalore 560064 India shobhana@jncsir.ac.in; Department of Chemistry, Yale University 225 Prospect Street New Haven Connecticut 06520 USA victor.batista@yale.edu; School of Materials Sciences, Indian Association for the Cultivation of Science Jadavpur Kolkata 700032 India camnp@iacs.res.in; Energy Sciences Institute, Yale University 810 West Campus Drive West Haven Connecticut 06516 USA; Department of Electrical and Computer Engineering, National University of Singapore Singapore 117583 Singapore; Department of Materials Science and Engineering, National University of Singapore Singapore 117575 Singapore

## Abstract

We demonstrate a strategy inspired by natural siderophores for the dissolution of platinum nanoparticles that could enable their size-selective synthesis, toxicological assessment, and the recycling of this precious metal. From the fabrication of electronics to biomedical diagnosis and therapy, PtNPs find increasing use. Mitigating concerns over potential human toxicity and the need to recover precious metal from industrial debris motivates the study of bio-friendly reagents to replace traditional harsh etchants. Herein, we report a family of redox-active siderophore-viz. π-acceptor azo aromatic ligands (L) that spontaneously ionize and chelate Pt atoms selectively from nanoparticles of size ≤6 nm. The reaction produces a monometallic diradical complex, Pt^II^(L˙^−^)_2_, isolated as a pure crystalline compound. Density functional theory provides fundamental insights on the size dependent PtNP chemical reactivity. The reported findings reveal a generalized platform for designing π-acceptor ligands to adjust the size threshold for dissolution of Pt or other noble metals NPs. Our approach may, for example, be used for the generation of Pt-based therapeutics or for reclamation of Pt nano debris formed in catalytic converters or electronic fabrication industries.

## Abstract 2

Biofriendly recycling of Pt is enabled by ligands that size-selectively dissolve nanoclusters.

## Graphical abstract

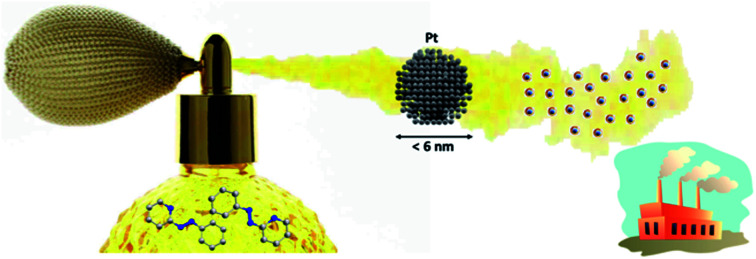

## Introduction

The use of platinum nanoparticles (PtNPs) as heterogeneous catalysts, fluorescent or colorimetric chemical sensors, food additives, antioxidant, microbial and cancer agents, as well as artificial enzymes has attracted enormous attention over the past few decades.^1–3^

More than 50% of the globally extracted Pt metal goes into the production of catalytic nanoclusters for a variety of chemical reactions in critical industrial processes such as catalytic conversion,^4^ cracking of crude oil,^5^ fuel cell redox reactions,^6,7^ hydrogen evolution reactions (HER),^8–10^ oxygen reduction reactions (ORR),^11,12^ and (de)hydrogenation reactions.^13–16^ In all these processes the chemical nobility of Pt remains uncompromised.

At the same time, while PtNPs have found numerous applications, concerns over how to recover the precious metal from catalytic converters or fabricated electronics, as well as concerns about toxicity from increased human exposure have emerged.^17^ The beneficial or detrimental physicochemical properties of PtNPs critically depend on their shape and size. PtNPs with diameters ≤6 nm were found to cause heptao- and genotoxicity, although the nature and mechanism of the size-dependent effects remain unclear.^1,17–19^ There is a pressing need to enable precise synthetic control over the size distribution of PtNPs to accurately assess and limit toxicity,^1^ and to realize the full potential of PtNPs in a variety of industrial and biomedical applications.^1,20^

Herein, we present a size-selective leaching and dissolution of PtNPs using redox active bio-inspired siderophores. Microorganisms use siderophores—low molecular weight chelators—to sequester iron from the environment, resulting in the weathering of minerals.^21^ In analogue to this process, we disclose a family of π-acceptor azo-aromatic ligands (L) that spontaneously and selectively chelate Pt atoms from sub-6 nm PtNPs within a polydisperse sample. The size selectivity of the approach, and the potential to tune the size threshold over a wide range distinguish the method from other oxidative etching strategies for the dissolution of noble metal nanoparticles,^22–24^ or other methods to sculpt nanoparticle morphology.^25^

Whereas natural siderophores extract already oxidized Fe^III^ from minerals, our synthetic siderophore-like chelators perform a two-electron oxidation of Pt^0^ resulting in a monometallic singlet diradical complex, [Pt^II^(L˙^−^)_2_] in high yield under ambient conditions.^26,27^ The mild reaction conditions are in stark contrast to the use of harsh reagents ordinarily required to overcome the chemical inertness of Pt. This finding may enable generation of Pt-based therapeutics, their toxicological assessment,^28–31^ and recycling of PtNPs in catalytic and electronic fabrication industries.

The size threshold for the dissolution reaction is shown by density functional theory (DFT) calculations to occur when the binding energy of the ligand for Pt^II^ is higher than (or at least, equal to) the sublimation energy of the PtNPs in the gas phase, and can be adjusted by modulating the electronic properties of the ligand. This knowledge builds upon current perspectives on metal–metal and metal–ligand interactions.^32–34^ We therefore add a versatile new tool to the canon of synthetic strategies for the size-selective preparation of PtNPs that can unleash their biomedical and technological potential. The strategy can also be used to recover Pt from catalytic converters and fabricated electronics through the dissolution of nanoparticles that fall within the size threshold. This approach moreover can be generalized to the preparation of other noble metal nanoparticles.

## Results

### Size selective dissolution of PtNPs by a π-acceptor azo-aromatic ligand

**Fig. 1:**
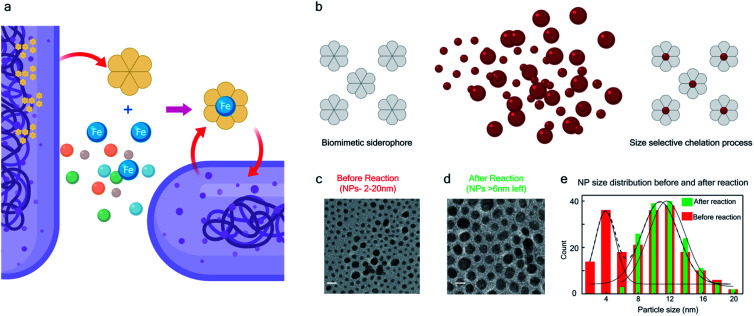
Bio-inspired siderophore-like etching of noble metal Pt – (a) schematic illustration of siderophore activity in a biological system. (b) The bio-inspired siderophore-like etching of Pt NPs by our π-acceptor azo aromatic ligand systems. (c) The TEM image of NP distribution (2–20 nm) before mixing with L. (d) TEM image showing that after mixing NPs (2–20 nm) with L, the NPs < 6 nm have been selectively dissolved, leaving only NPs > 6 nm behind. (e) The distribution of NP size, before and after the reaction.

The bio-inspired basis for our chemistry is schematically illustrated in Fig. 1a and b. In Fig. 1a, a chelator released by a microorganism reacts with iron in the ore, chelating the element from its host matrix. A similar effect is reproduced in Fig. 1b whereby the 2-(phenylazo)pyridine (L_1_), a member of the 2-(arylazo)pyridine family (L), reacts selectively with the surface Pt atoms on the nanoparticles with sub-6 nm diameters, ultimately leading to the dissolution of the NPs. The mechanisms of the Fe and Pt chelators are different and will be discussed later in this report.

The siderophore-like reactivity of the L_1_ ligand shows a size-selectivity that is absent from the natural counterparts. Upon mixing a polydisperse sample of PtNPs having a size distribution ranging from 2 to 20 nm (Fig. 1c–e) in a chloroform solution of L_1_, sub-6 nm PtNPs were dissolved, as determined by TEM analyses (Fig. 1e and S1–S8†). The color of the solution also changed from red to greenish brown (see the insets to Fig. S2a and b†). In contrast, nanoparticles with size >6 nm remain unreacted, suggesting the possibility of a colorimetric assay for nanoparticle size. These findings were further confirmed by exposing approximately mono-disperse nanoparticle samples with sizes ≤6 nm (Fig. S2 and S4†) and >6 nm (Fig. S2 and S5†). PtNPs were quantitatively dissolved in the former sample but remained intact in the latter sample. Control experiments were also performed with different batches of NPs (Fig. S8†), different NP morphology (Fig. S3†), different solution concentration (Fig. S8†) all yielding the same size threshold of ∼6 nm. To further verify the robustness of estimation of this size threshold, a mixture of the NPs > 6 nm with L_1_ was stirred continuously for 3 days and even then, no reaction occurred confirming that the estimation of the size threshold of ∼6 nm is reliable.

Because Pt as a noble metal is ordinarily chemically inert, except under treatment by harsh reagents like aqua regia or fluorine gas, we sought to understand the mechanism of the size-selective reactivity. We used time-resolved nuclear magnetic resonance (NMR) and Visible-NIR absorption spectroscopies to monitor the electronic interactions between the NPs (with *d* ≤ 6 nm) and the ligand (L_1_) in a polydisperse mixture of PtNPs during the reaction.

**Fig. 2:**
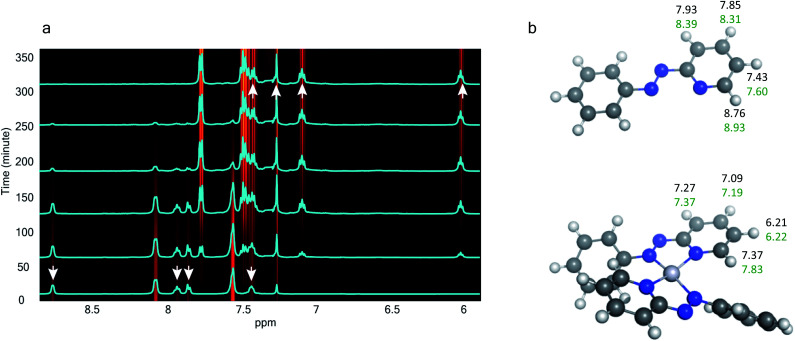
*In situ* time resolved ^1^HNMR spectroscopy – (a) temporal evolution of ^1^HNMR spectra. Spectra obtained at six selected times are overlaid on the pseudo-color map. (b) Assignment of experimental ^1^HNMR resonances to the free azo-aromatic reactant and the monometallic coordinated product based on DFT computations. The structures were optimized with B3LYP/SDD for Pt; 6-311G(d,p) for H, C, and N. The same functional with the def2TZVP basis set was used to simulate the ^1^HNMR properties in an implicit chloroform solvent.

The ^1^H NMR spectra taken at six different times (*t* = 10, 55, 130, 200, 275, 325 min) are plotted in blue and overlaid on a pseudo-color plot of NMR (Fig. 2a) generated from all the spectra taken at *t* ∼ 30 min intervals (see the Methods section for experimental details). We observe that at *t* = 0 min, the ^1^HNMR spectrum displays pyridyl proton resonances at *δ* = 8.76, 7.93, 7.85 and 7.43 ppm (Fig. 2a), which are assignable to the free ligand by DFT computations (Fig. 2b).^35^ With time, these peaks get gradually shielded and shifted to higher fields, indicating accumulation of negative charge on the pyridyl ring. At *t* > 200 min, well-resolved pyridyl proton resonances start appearing at *δ* = 6.21, 7.09, 7.27 and 7.37 ppm. These resonances, particularly the most up field ones, are diagnostic for the formation of a metal coordinated complex of the azo-anion radical ligand 
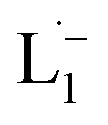
 according to our DFT calculations (Fig. 2b) and literature precedents.^36^ Notably, in the final ^1^HNMR spectrum, aromatic proton resonances for nine protons are observed, suggesting that the two coordinated ligands in 
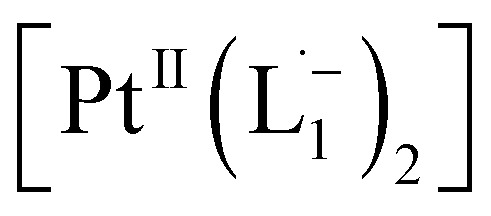
 are magnetically equivalent. The structure of this PtNP dissolution product is characterized with a suite of experimental and computational methods in the following section and ESI.†

**Fig. 3:**
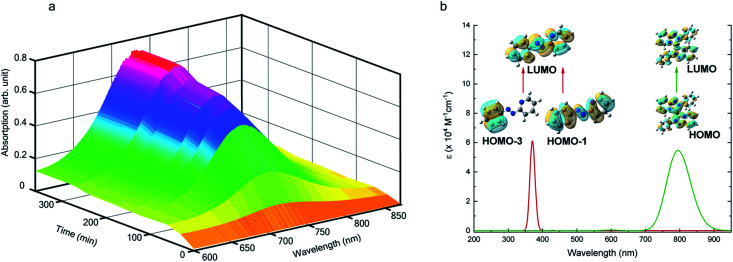
*In situ* UV-Vis-NIR spectroscopy – (a) 3D-surface plot of the evolution of Visible-NIR spectra with time showing a temporal growth of an absorption peak at 725 nm. (b) The DFT computed spectra showing two absorption peaks at 795 nm and 370 nm. In (a), we only show the traces from 600 nm as the lower wavelength features are heavily dominated by polyethylene glycol (PEG) which is used to stabilize the NPs.

We also followed the reaction with UV-Vis-NIR spectroscopy where the spectra were collected from the solution at different points in time after mixing, ranging from 0 to 500 min. A three-dimensional surface plot of the time-resolved UV-Vis-NIR spectra is presented in Fig. 3a (also see Fig. S9–S12†). Simulated spectra for free L_1_ and the 
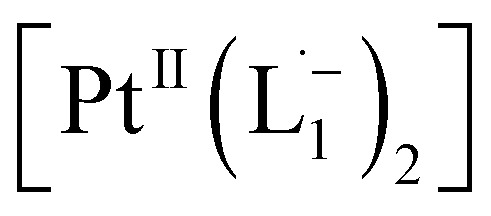
 complex are also shown in Fig. 3b with the corresponding orbital transitions depicted. The computed spectra allow an unambiguous identification of the absorption peak at 725 nm experimentally (795 nm computationally). Note that in the calculated spectrum there is a peak at 370 nm, which can be seen in an isolated [Pt^II^(L˙^−^)_2_] complex (Fig. S13†) but does not appear in the *in situ* measurement. This is because, in the data presented in Fig. 3a, the spectral features less than 600 nm are heavily dominated by polyethylene glycol (PEG), which is used to stabilize the NPs.

With progress in reaction time, the growth of an absorption peak at 725 nm (1.65 eV) is observed, which is in excellent agreement with the predicted absorption for 
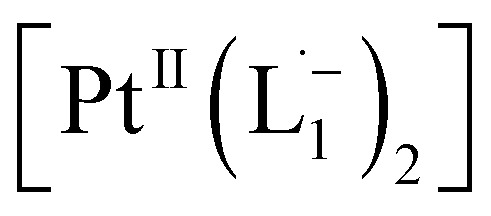
 at 795 nm (1.56 eV). Verified with DFT calculations, this absorption feature arises from the π to π* (HOMO to LUMO) transition (Fig. 3b) in Pt-complexes^37^ of radical ligands. The inset to Fig. 3b indicates that d-orbitals of the central Pt atom participate in the photoexcitation. Consistent with the results from the *in situ* NMR studies, the main changes in the Visible-NIR spectra are also observed to occur between *t* = 0 and 350 min. Beyond *t* = 360 min, the spectral trend does not exhibit any change, indicating the formation of a stable product.

We isolated a stable product from the above chemical reaction and characterized it with several physical techniques including X-ray photoelectron spectroscopy (XPS), Raman spectroscopy, mass spectrometry, and single crystal X-ray crystallographic data, alongside results from DFT calculations, we could confirm the formation of a 
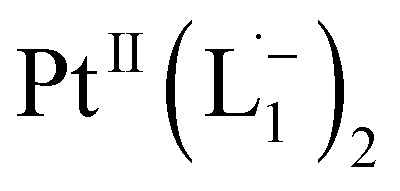
 complex (discussed in the following section). Notably, both ^1^H NMR and UV-Vis-NIR spectra measured at *t* > 360 min (Fig. 2 and 3) show an excellent match with the spectrum of the isolated product confirming the formation of the 
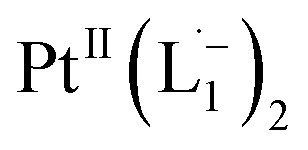
 complex in the *in situ* measurement process (Fig. S9–S14†). Additionally, Fig. S9 and S10† indicate that the intermediate UV-Vis-NIR or NMR spectra can be expressed as a linear combination of the initial and final spectra which enables us to use the *in situ* data to estimate the reaction rate (Fig. S11 and S12†). This implies a gradual etching process consistent with the scheme presented in Fig. 5c and discussed below: two molecules of L_1_ combine with a Pt atom on the surface of a sub-6 nm Pt^0^ nanoparticle to ionize (by 2-electrons) and chelate Pt out of the nanoparticle forming the diradical-complex, 
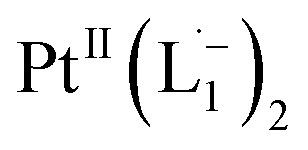
. To verify this picture of sequential etching, we stopped the etching process in between, by adding 1 equivalent of L_1_, less than the stoichiometric quantity of 2 equivalents as shown in Fig. S7,† this resulted in an incomplete etching of the NPs yielding smaller sized particles than the starting sample.

**Fig. 4:**
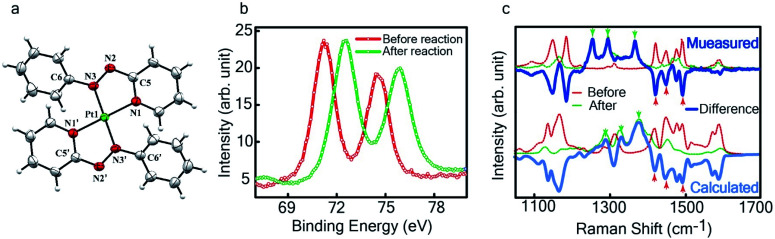
Characterization of the isolated product – (a) three-dimensional molecular structure of the complex formed by the reaction between Pt NPs (<6 nm) and L_1_, determined from X-ray diffraction analysis of an isolated crystal. (b) XPS of the drop cast film of Pt^0^-NPs and isolated 
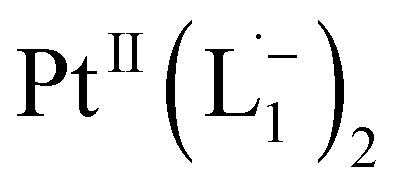
 (c) Raman spectra of the drop cast films before and after the reaction (dotted lines) and their difference (in solid blue). The calculated spectra obtained by subtracting the DFT generated spectrum for L_1_ from that of 
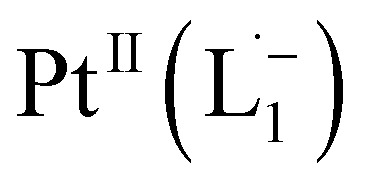
 a reasonable match to the experimental data for the differential spectrum.

**Fig. 5:**
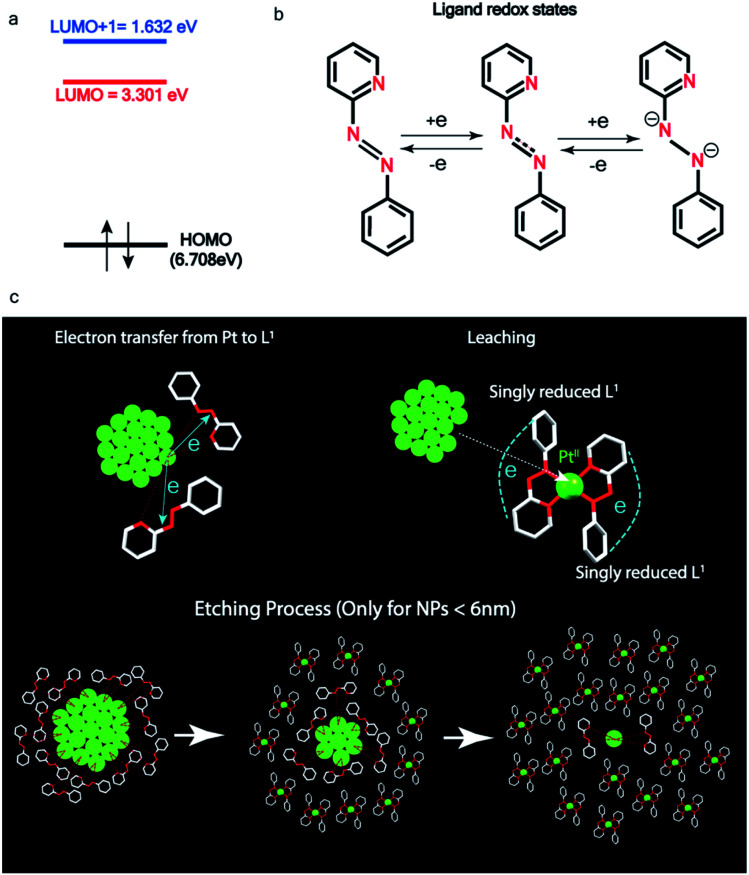
Pt ionization and etching – (a) calculated redox potential of L_1_ with respect to vacuum and (b) corresponding ligand redox states. (c) Schematic illustration of ionization and etching of Pt.

### Product isolation/characterization

The single crystal X-ray crystallographic analysis of the isolated, dark green product (Tables S1–S2†) unambiguously revealed the formation of the compound 
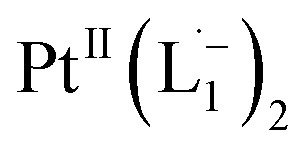
, having a square planar structure with crystallographic *C*_2_-symmetry. This is very much consistent with the fact that Pt has a stable valence of +2 and the coordination number of Pt^2+^ is four, allowing it to form a coordination complex hosting two bidentate ligands. The Oak Ridge Thermal Ellipsoid Plot (ORTEP) and atom-numbering scheme (selected atoms only) of this complex are shown in Fig. 4a. The two azo-anion bonds of the coordinated ligands are identical with *d*_N–N_ = 1.335(5) Å; this is longer than that in a coordinated neutral L_1_ (where *d*_N–N_ = 1.258(5) Å, as reported^38^ in PtCl_2_(L_1_)). Moreover, the Pt–N(azo) bond-length in this complex is 1.965(3) Å, which is smaller than the Pt–N(py) bond length (2.006(3) Å) and indicates the accumulation of negative charge on the coordinated azo-functions (consistent with the observations in ^1^H NMR and Vis-NIR spectra).

To determine the change in Pt-oxidation state before and after the reaction, we used X-ray photoelectron spectroscopy (XPS). As shown in Fig. 4b, before the reaction, we obtained peaks at 71.2 eV (4f_7/2_) and 74.5 eV (4f_5/2_), corresponding to the Pt(0) state,^39,40^ whereas after the reaction the peaks shifted to 72.52 eV (4f_7/2_) and 75.89 eV (4f_5/2_), which are characteristic^39–41^ of Pt^II^. This spectral shift confirms that the Pt atoms from the nanoparticle surface undergo two-electron oxidation in the L_1_ environment, strongly corroborating^36^ the identification of a Pt^II^-diradical complex of the azo-anion ligand, 
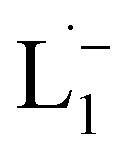
.

To further characterize the electronic description of the isolated complex, we performed structural optimization using DFT. The metrical parameters, calculated for 
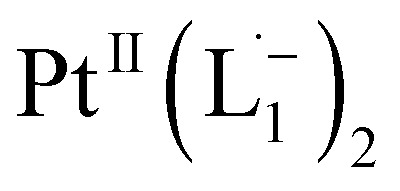
, are in good agreement with the experimental values (Table S2†). The spin density plot of 
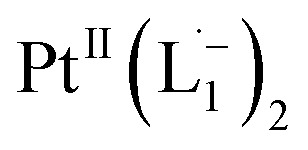
, as obtained using DFT, is shown in Fig. S15.† By analyzing our DFT results, we found that in the LUMO and LUMO+1 (electron acceptor) orbitals, 73% and 58% of the charge density is localized on the azo group. Hence, we monitored the azo-vibrational modes in the samples using Raman spectroscopy to assess the ligand redox state.

In Fig. 4c, we show the differential Raman data, obtained from the spectra recorded before and after the reaction, and contrast these with the corresponding calculated spectra. Before the reaction, the vibrational peaks of the azo-modes (indicated by the red arrows in Fig. 4c) are observed at 1493, 1450 and 1423 cm^−1^, whereas post-reaction, azo-vibrational modes occur at 1367, 1295, and 1255 cm^−1^ (indicated by the green arrows). The experimentally obtained differential spectra show a reasonably good match to differential vibrational frequencies obtained by simulating Raman spectra of L_1_ and 
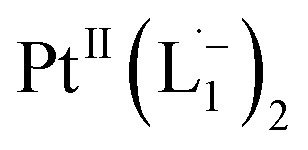
 using DFT calculations (L_1_: 1570, 1497, 1478 cm^−1^; 
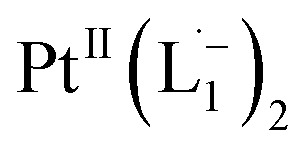
: 1434, 1351 and 1306 cm^−1^), validating the formation of the diradical complex.

Based on all the above results, we unambiguously conclude that for nanoparticles of size ≤6 nm, individual Pt atoms are 2-electron ionized and etched from the nanoparticle by the L_1_ ligand, resulting in the formation of the diradical complex, 
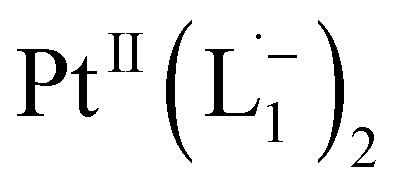
.

## Discussion

### Mechanism

Ionization and etching of Pt nanoparticles is completely contrary to the expected chemical inertness and sintering behavior of Pt. However, the experiments described in the preceding sections suggest the following reaction scheme.

The ligand L_1_, owing to its strong electron-affinity, dresses the NPs (which are a pool of electrons) in solution. L_1_ (or in general L) is characterized by a low-lying redox energy level (Lowest Unoccupied Molecular Orbital – LUMO at 3.301 eV) making it a strong π-electron acceptor that can readily accept up to two electrons (see Fig. 5a and d). The ligand ionizes the outer surface atoms of the NPs < 6 nm and chelates them in the form of a complex, 
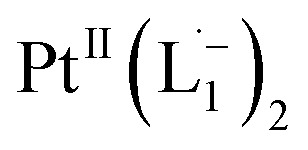
 (characterized above). This etching of the NPs is visualized in Fig. 5c. The process exposes the next inner layer of the atoms of the NP to the ligands and the dissolution continues until each atom in the cluster is ionized and etched by L_1_ (see Fig. 5c). Eventually, the process leads to a complete dissolution of the entire NPs – this is very different from the usual catalytic activity of Pt-NPs, where only the surface atoms participate^42^ and remain bound to the cluster. The process is instead reminiscent of the dissolution of minerals by microorganisms that scavenge and sequester iron *via* the use of siderophores. Whereas a usual siderophore does not involve electron transfer, here the ligands cause a 2-electron ionization of the Pt atoms.

In order to assess the reaction scheme, we performed DFT calculations (Fig. S16–S18 and Tables S3–S4†) where we considered the total energy of N Pt atoms as a function of nanoparticle size *n*, for a sequential process where one Pt atom is either added to a Pt_*n*_ cluster to form a Pt_*n*+1_ cluster (sintering) or detached from it to form a Pt_*n*−1_ cluster (etching). The energetics of these two processes were compared in the gas phase and in the ligand-environment, where L_1_ ligands are available to bind to Pt atoms.

**Fig. 6:**
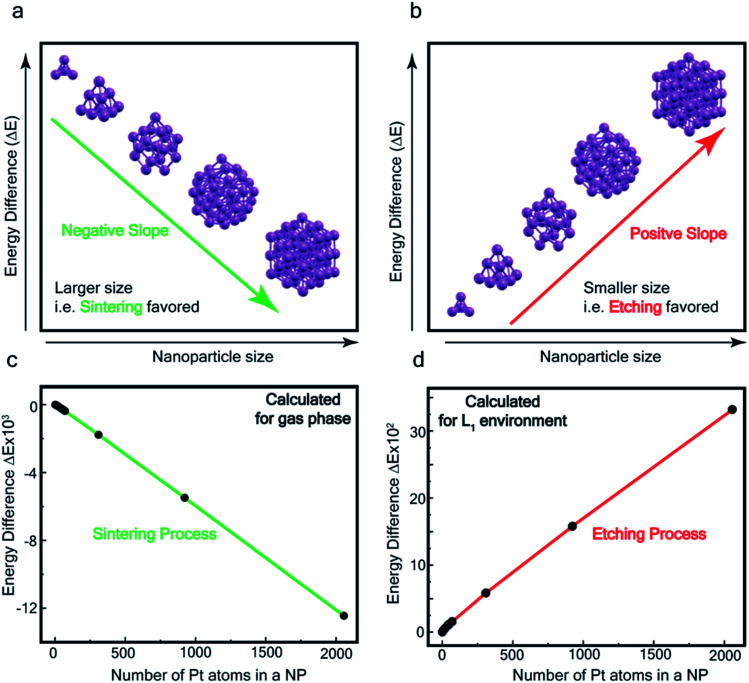
Theoretical calculation – (a and b) illustration of the slope of energy-difference (Δ*E*) *vs.* NP size (*n*) (*i.e.* δ(Δ*E*)/δ*n*) for (a) sintering (agglomeration energy) and (b) etching (dissociation energy). Calculated Δ*E vs. n* plot in (c) gas phase and (d) L_1_ environment.

The processes of sintering and etching are schematically illustrated in Fig. 6a and b. If sintering was thermodynamically favored, the slope of the graph of energy difference *vs. n* (*i.e.* δ(Δ*E*)/δ*n*) would be negative (see Fig. 6a) and, in contrast, if etching was favored, the slope would be positive (see Fig. 6b). Fig. 6c and d compare the DFT data, obtained for the gas phase and L_1_ environment (where *n* is the number of Pt atoms, shown on the *x*-axis, to form a Pt_*n*_ nanoparticle and the remaining (*N*–*n*) Pt atoms are in the gas phase). Notably, the slope of Fig. 6c is negative, whereas it is positive in Fig. 6d. This indicates that, while the L_1_ environment facilitates etching, the usual gas phase favors sintering. Note that because of the high computational cost, we could perform DFT calculations only up to *n* = 2057, beyond which, results are obtained by a quadratic extrapolation (Section-S4 and Fig. S17†).

**Fig. 7:**
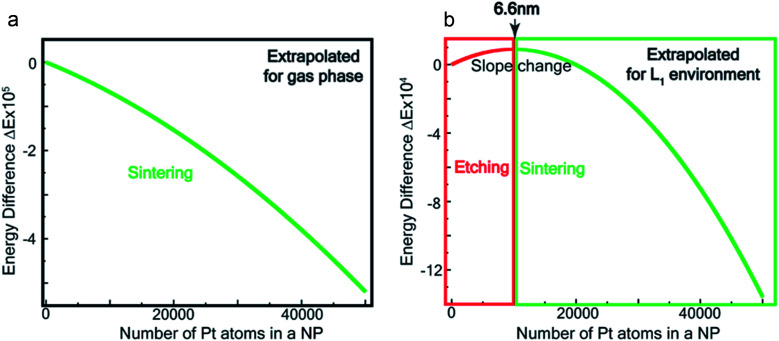
Theoretical estimation of size threshold –extrapolated Δ*E vs. n* plot (see ESI† for details) for (a) gas phase and (b) L_1_ environment.

Fig. 7a and b show the extrapolations to a particle size *n* = 50 000 (note that the zero of the ordinate is chosen to occur for *n* = 1). In the gas phase the extrapolated slope of the graph (*i.e.* δ(Δ*E*)/δ*n*) is always negative, as expected, for the entire range of *n* (Fig. 7a), *i.e.* sintering is thermodynamically favored. In contrast, in the ligand environment (Fig. 7b and Section S4†) the extrapolated (δ(Δ*E*)/δ*n*) (slope) starts out strongly positive at *n* = 1, but decreases until reaching a threshold size *n*^th^. Beyond this threshold, the slope is negative. The value of *n*^th^ in the extrapolated curve can be estimated as 9930 (*d* ∼ 6.6 nm) with a fitting, that yields a correlation coefficient of 99.98%. This implies that below n, etching is favoured, whereas above this value, NPs tend to sinter, which is in close agreement to the experimentally observed values, where we found that the reactions occurred only for nanoparticles with *d* ≤ 6 nm. This also supports our experimental observation that larger nanoparticles tend to agglomerate (sinter) when mixed with L1, as can be seen from the TEM image shown in Fig. S5† which is also known as Ostwald Ripening. It is important to note that here the etching of NPs > 6 nm is thermodynamically prohibited and hence is not limited by kinetic barriers. This is why changes in factors like etching time (or rate), ligand concentration or morphology do not have an effect on the size dependence of this reaction. For smaller NPs (<6 nm) however, where the reaction is thermodynamically favoured, varying factors like concentration does change the reaction rate (see Fig. S17†).

We show in the ESI (Section-S4, Fig. S16 –S23, Table S3 and S4†) that in the presence of the ligand family L (L_1_ is a member of that family, Fig. S16†), the maximum (or threshold) size where the etching stops (*i.e. n*^th^) is given by *E*_sub_(*n*^th^) = *E*_b_
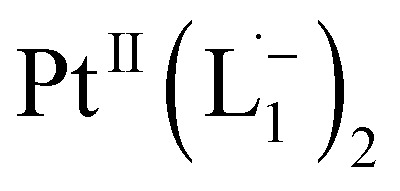
. Here *E*_sub_(*n*) is the sublimation energy for a Pt nanoparticle of size *n* in the gas phase, and *E*_*b*_
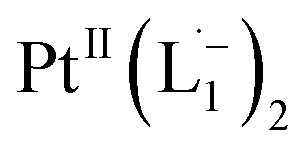
 is the binding energy of the 
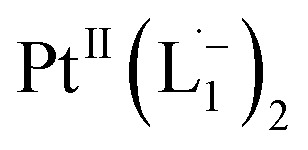
 complex. We note that *E*_sub_*(n)* is, in general, an increasing function of *n*. Thus, by changing the ligand L_1_ to other ligands with a smaller/larger binding energy to the Pt atom, the critical size at which the etching stops would also become smaller/larger. This tenability of the ligand system has been verified with a series of ligands, shown in Section-S4.† This result opens up the possibility of designing and engineering different ligand systems to come up with different size threshold values for etching.

A further implication of our experiments is the use of PtNPs as a starting material for designing anion radical complexes in high isolated yields. In fact, this turns out to be a much more efficient synthetic route compared to a conventional approach of starting from a chloride salt of Pt. We attempted the synthesis of Pt(L_1_)_2_*via* a reaction between PtCl_2_(L_1_) and L_1_ in the presence of a reducing agent, NEt_3_. The reaction produced Pt(L_1_)_2_ (<2% yield) merely as a contaminated product with a major insoluble mass of undefined composition. The use of reducing alkyl amines in this chemical process triggered the idea of forming PtNPs as intermediates, prompting us to use pre-formed and stabilized Pt-NPs as starting reactants. This approach resulted in >75% yield of the pure product that could be isolated in the form of single crystals. Similar strategies might be useful for significantly improving the synthesis of other noble metal complexes that are known to suffer from a low yield.

However, it should be noted that a confluence of several factors enable this reaction, such as, (a) energy orientation of the participating orbitals favoring the electron transfer from the metal atoms in a NP to the ligands, (b) the higher enthalpy of the metal–ligand bond compared to the dissociation energy of a Pt-atom form the NP, (c) the coordination number 4 of Pt^II^ supporting the 2 bidentate ligand coordination to the metal center and (d) the air stability of the complex. All these conditions need to co-exist to support this reaction and these should be taken into consideration for exploring other metal and ligand systems.

## Conclusions

To summarize, we demonstrated siderophore-like reactivity of azo-aromatic ligands that spontaneously and size-selectively dissolve PtNPs forming monometallic Pt(ii) complexes. These azo-aromatic ligands can therefore be applied to decrease the polydispersity of a NP preparation, to prepare Pt-based therapeutics as PtNPs of suitable size (>6 nm) or the [Pt^II^(L˙^−^)_2_] complexes resulting from NP dissolution, and to detect PtNPs as a size-selective colorimetric assay. Furthermore, our computations indicate that the size threshold for NP dissolution can be adjusted over a considerable range by engineering the ligand to change its binding energy to the metal. Given that Pt has an unusually high cohesive energy relative to other metals, our bio-inspired method is likely applicable for other transition metals, offering a one-pot, high yield chemical synthesis of size-selected NPs or radical complexes, which usually involve multistep synthetic routes.

The inability to dissolve Pt poses several long-standing industrial challenges at present, several of which can be resolved based on the results presented here. Transition metal complexes of azo-aromatic ligands are, in general, gaining momentum for their possible application for the next generation of electronics.^43–46^ One pot synthetic routes of stable radicals can be of benefit for such applications. Other examples include the dissolution of nano-debris that is unavoidably formed during electronic fabrication and contributes to the malfunctioning of circuits. Our size selective dissolution strategy offers a way to dissolve only the debris without affecting the larger sized electrodes. Additionally, the reaction presented here offers a technique to recycle precious noble metal particles from industrial debris (such as in catalytic converters). The organic parts of the Pt-complex formed by dissolution of PtNPs in L could be evaporated by plasma treatment,^47–49^ leaving behind the metal for recovery.

## Materials and methods

### Synthesis of ligands

The ligands 2-(phenylazo)pyridine (L_1_), 2-(4-chlorophenylazo) pyridine (L_2_), 2-(4-methylphenylazo)pyridine (L_3_) and 2-(phenylimino)pyridine (Λ_2_) were prepared as reported previously,^35,50–52^ whereas the ligands 9,10-phenanthroline (Λ_1_) and 2, 2′-bipyridine (Λ_3_) were purchased from Sigma-Aldrich.

### Synthesis of platinum nanoparticles (size range: 1–6 nm), group 1

In a 50 mL three-neck round bottom flask, 0.28 g of K_2_PtCl_4_ and 1.82 g of CTAB (Cetyl Trimethyl Ammonium Bromide) were dissolved in 15 mL of de-ionized water by constant stirring at room temperature. The clear solution was placed in an ice bath to maintain the temperature between 0–4 °C. In a separate vial, 3 mmol (0.113 g) of NaBH_4_ was dissolved in 5 mL ice-cold and deionized water. Then the solution was added dropwise to the parent mixture over a period of 5 min with constant stirring. The colour of the solution changed from yellow to brown and the mixture was stirred for another 30 min. Finally, the brown Pt nanoparticles were washed with ethanol and harvested in chloroform/polyetheneglycol (PEG) mixture.

### Synthesis of platinum nanoparticles (size range: 7–12 nm), group 2

Medium size nanoparticles (7–12 nm) were prepared following a reported^53^ procedure. Under a nitrogen flow, 0.2 g of Pt(acac)_2_ (acac = acetylactonate) was mixed with 10 mL 1-octadecene (ODE), 1 mL oleic acid and 1 mL oleyl amine. The mixture was slowly heated to 400 K in 20 min. After 30 min of heating, one drop (0.01–0.03 mL) of Fe(CO)_5_ solution (prepared by dissolving 0.1 mL Fe(CO)_5_ in 1 mL ODE) was added to this solution. The temperature was then raised to 473 K (3–5 °C min^−1^), which was maintained for 30 min. The solution was cooled down to room temperature. On addition of 40 mL of isopropanol to the mixture, the product was separated by centrifugation (8000 rpm for 10 min), which was then dispersed in hexane.

### Synthesis of platinum nanoparticles (size range: 2–20 nm), group 3

It was synthesized following the same procedure as described above for group 1 with only exception that NaBH_4_ solution was injected at 320 K instead of slow addition of its cooled solution. Upon cooling down the mixture to room temperature, Pt nanoparticles having 2–20 nm range were obtained.

0.1 mL solution of the mixture (group 1–3) were diluted further with 0.5 mL methanol. The size distribution of the nanoparticles, synthesized as above, were determined by examination of their TEM images.

The amount of Pt-NPs were calculated based on the quantity of precursor used. Herein we have used 1 mmol of K_2_PtCl_4_/Pt(acac)_2_ to synthesize 1 mmol of Pt-NPs (contains 1 mmol Pt-atoms) which subsequently reacted with 2 mmol of the ligand.

### Synthesis of Pt(L^−^˙)_2_ complexes from group 1 nanoparticles

#### Reaction with L_1_

1.0 mmol of preformed group 1 nanoparticles were allowed to react with 2.0 mmol (0.366 g) of the 2-(phenylazo)pyridine ligand (L_1_) in chloroform solvent for 6 h. The color of the solution became dark brown. The mixture was then evaporated under vacuum and the crude mass was thoroughly washed with water to remove polyethylene glycol. A dark mass, thus obtained, was dried and dissolved in dichloromethane (5 mL). The compound was purified *via* rapid precipitation from dichloromethane solution of it by excess addition of hexane. Single crystals of 
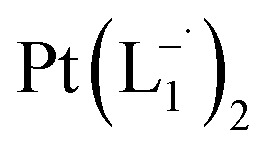
 suitable for single crystal X-ray diffraction analysis were obtained by slow diffusion of its dichloromethane solution into hexane. Isolated yield: (75%, 0.42 g). ESI-MS, *m/z*: 561.96 for [Pt(L_1_)_2_]^+^. Anal. calcd for C_22_H_18_N_6_Pt: C, 47.06; H, 3.23; N, 14.97. Found: C, 46.85; H, 3.19; N, 14.89. ^1^HNMR (500 MHz, CDCl_3_)*δ*: 6.03 (t, 1H, *J* = 6.5 Hz), 7.09 (m, 1H), 7.28 (m, 1H), 7.35–7.45 (m, 4H), 7.72 (d, 2H, *J* = 7.5 Hz).^13^CNMR (125 MHz, CDCl_3_) *δ* 112.87, 117.76, 126.52 (2C), 127.75, 129.87 (2C), 133.09, 139.43, 145.47, 149.54. Similar synthesis also can be performed using methanol as solvent.

#### Reactions with L_2_ and L_3_

The complexes 
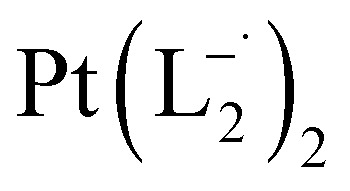
 and 
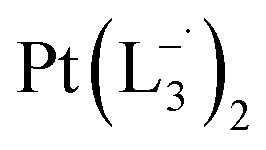
 were synthesized following an identical procedure as it is for 
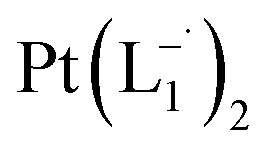
, using the ligand L_2_ and L_3_ respectively in place of L_1_.

#### Characterization data for 
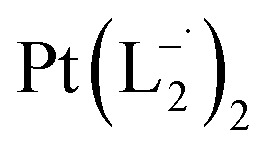


(Isolated yield: 78%). ESI-MS, *m*/*z* 629.04 for 
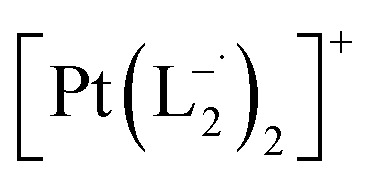
 anal. calcd for C_22_H_16_Cl_2_N_6_Pt: C, 41.92; H, 2.56; N, 13.33. Found: C, 41.88; H, 2.52; N, 13.30. ^1^HNMR (400 MHz, CDCl_3_)*δ*: 7.03 (m, 1H), *δ* 7.17 (d, *J* = 8.0 Hz, 2H), *δ* 7.46 (d, *J* = 8 Hz, 1H), *δ* 7.53 (m, 1H), *δ* 7.71 (d, *J* = 8 Hz, 2H), *δ* (d, *J* = 7.2 Hz, 1H). ^13^CNMR (100 MHz, CDCl_3_) *δ*: 114.85, 124.18(2C), 125.10, 128.85(2C), 137.47, 138.04, 148.86, 150.06, 161.90.

#### Characterization data for 
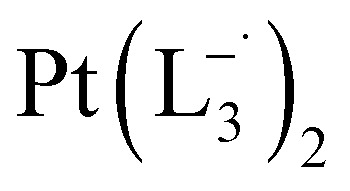


(Isolated yield: 75%) ESI-MS, *m*/*z* 589.54 for [Pt(L_3_)_2_]^+^ anal. calcd for C_24_H_22_N_6_Pt: C, 48.89; H, 3.76; N, 14.25. Found: C, 47.25; H, 3.78; N, 14.18. ^1^HNMR (400 MHz, CDCl_3_)*δ*: 7.59 (d, *J* = 8.0 Hz, 2H), *δ* 7.383 (d, *J* = 8.0 Hz, 1H), *δ* 7.33 (d, *J* = 7 Hz, 1H), *δ* 7.24 (d, *J* = 8.0 Hz, 2H), *δ* 7.05 (t, *J* = 8 Hz, 1H), *δ* 6.03 (t, *J* = 7.0 Hz, 1H). ^13^CNMR (100 MHz, CDCl_3_) *δ*: 146.06, 143.12, 137.97, 132.81, 130.40(2C), 127.75, 126.47(2C), 117.50, 114.23, 29.85.

#### Reaction with group 2 nanoparticles

The reactions with group 2 nanoparticles were carried out similarly as above. However, none of 2(arylazo)pyridines ligands (L_1–3_) reacted.

Similar reactions with group 1 nanoparticles with all three ligands of Λ-series (Λ_1–3_) failed to react. The stirring in these cases was continued for 24 h.

#### The reaction of L_1_ with group 3 nanoparticles

Similar reaction protocol was employed with group 3 nanoparticles. In this case, only the Pt-NPs with a size distribution ranging between 1–6 nm got dissolved, while NPs ranging between 7–20 nm remained unreacted as detected by TEM analysis (Fig. 1d).

### Control experiments

#### The reaction of one equivalent L_1_ with group 1 nanoparticles

1.0 mmol of preformed group 1 nanoparticles were allowed to react with 1.0 mmol (0.183 g) of the 2-(phenylazo)pyridine ligand (L_1_) in chloroform solvent for 6 h. The color of the solution became dark brown. The reaction was drop casted on a Cu-grid for post reaction TEM analysis which indicates an overall decrease in the size of Pt-NPs. The formation of the desired 
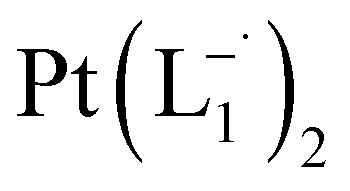
 complex was characterized by UV-Vis-NIR analysis.

#### The reaction of six equivalent L_1_ with group 3 nanoparticles

1.0 mmol of preformed group 3 nanoparticles were allowed to react with 3.0 mmol (0.549 g) of the 2-(phenylazo)pyridine ligand (L_1_) in chloroform solvent for 10 h. The color of the solution became dark brown. The reaction was drop casted on a Cu-grid for post reaction TEM analysis which indicates complete dissolution of Pt-NPs of size <6 nm. Post reaction size distribution analysis was depicted in Fig. S8.† The formation of the desired 
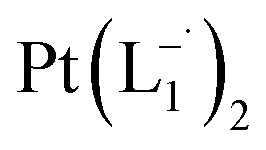
 complex was characterized by UV-Vis-NIR analysis.

#### *In situ* absorption spectral measurements to follow the formation of 
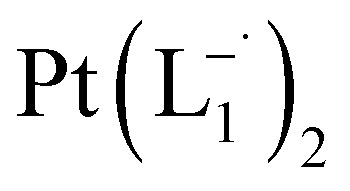


10 mL 0.02 molar solution of group 1 nanoparticles in chloroform was diluted to 100 mL by methanol to prepare a 0.002 molar stock solution of Pt-NPs. Another 10 mL 0.004 molar stock solution of 2-(phenylazo)pyridine (L_1_) was prepared in methanol. In a typical experiment, 10 mL stock solution of the nanoparticles was mixed with 10 mL stock solution of L_1_ maintaining the ratio of Pt-NPs : L_1_ = 1 : 2. 2 mL of the mixture solution was transferred to a cuvette and allowed to stir at room temperature (300 K). Spectra (in the range, 500–1200 nm) were recorded at an interval of 30 min. The growth of absorption at ∼730 nm in the spectrum was followed as a function of time.

Similar experiments were carried out with L_2_and L_3_ to follow the formations of 
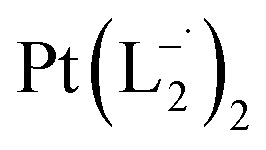
 and 
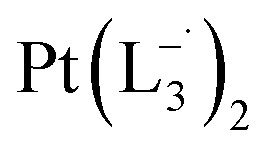
, respectively.

The relative rate of formation of Pt(L^−^˙)_2_was found to be different. For example, the formation of 
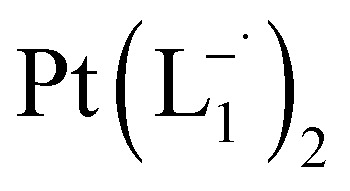
 is 2.14 time slower than that of 
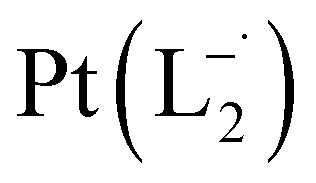
 but 3.6 time faster than that of 
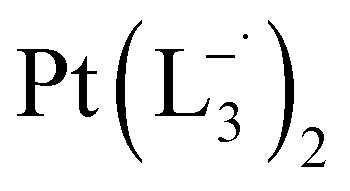
 (see ESI Appendix, Fig. S19†).

#### *In situ* ^1^H NMR spectroscopic study to follow the formation of 
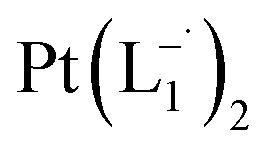


In a properly capped NMR tube, a CDCl_3_ solution containing 0.2 mmol of L_1_ ligand was mixed with an 0.1 mmol amount of group 1 nanoparticles and the reaction was monitored using time-dependent ^1^HNMR spectroscopy following 30 min time interval (Fig. 2a and S8†).

#### Materials and instrumentation

K_2_PtCl_4_was purchased from Arora-Matthey, Kolkata. All other chemicals were of reagent grade and used as received. Solvents were purified and dried before use.

A PerkinElmer Lambda 950 spectrophotometer was used to record UV-Vis-NIR spectra. Infrared spectra were obtained using a PerkinElmer 783 spectrophotometer. A PerkinElmer 240C elemental analyser was used to collect microanalytical data (C, H, N). ESI mass spectra were recorded on a micro mass Q-TOF mass spectrometer (serial no. YA 263). ^1^HNMR spectra were taken on a Bruker Avance 400 and 500 spectrometers. Room temperature magnetic moment measurement for 
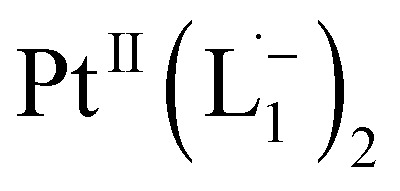
was carried out with a Gouy balance (Sherwood Scientific, Cambridge, UK). TEM and high-resolution TEM images were taken on a UHR-FEGTEM, JEOL JEM 2100 F and JEOL JEM1400 model using a 200 kV electron source. Specimens of the samples were prepared by dropping a purified nanoparticles solution in methanol on a carbon-coated copper grid, and the grid was dried in air. XPS spectra of the samples were measured in ultra-spectrometer under monochromatic Al Kα irradiation (180 W) at room temperature (300 K). Chloroform solution of the samples was drop cast on a glass surface and dried for 36 h under vacuum. A low-energy electron gun was used to compensate for the surface charge. At least two replicate measurements were carried out at a pressure of about 10^−9^ Torr. The XPS spectra were referenced to the C–C/C–H component of the C_1s_ peak of the samples and assumed to have the binding energy of 285.0 eV. A Bruker SMART APEX-II diffractometer equipped with graphite-monochromated Mo Kα radiation (*λ* = 0.71073 Å) was used for X-ray data collection.

#### Computational methods

Our calculations have been performed within the framework of *ab initio* spin polarized density functional theory, using the Quantum ESPRESSO (QE), SIESTA and Gaussian packages. A combination of packages is used because different codes have different capabilities regarding which properties can be easily calculated. In all cases, exchange-correlation interactions are treated using the PBE form for the generalized gradient approximation. Van der Waals interactions are incorporated using the DFT-D2 method. In QE, the Kohn–Sham equations are expanded using a plane wave basis set, with cut-offs of 40 Ry for wavefunctions and 400 Ry for charge densities. Interactions between the ionic cores and valence electrons are described using ultra soft pseudopotentials. In SIESTA, we use a double-*z* polarized localized basis with a mesh cutoff size of real-space grid taken to be 200 Ry. The Raman and UV-Vis spectra of the ligand L and the 
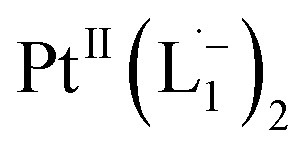
 complex have been computed using the Gaussian package, using 6-311G(d,p) basis for lighter atoms (C, H and N) and SDD basis for Pt atoms, along with B3LYP exchange-correlation functional. Vertical electronic excitations based on B3LYP optimized geometries were computed using the time-dependent density functional theory (TD-DFT) formalism in acetonitrile using conductor-like polarizable continuum model (CPCM). GaussSum was used to calculate the fractional contributions of various groups to each molecular orbital. In all cases, a vacuum spacing of at least 10 Å is introduced along with all non-repeating directions and the reciprocal space sampling is done at the zone center only.

## Author contributions

DS and Sreebrata Goswami (SG1) devised the project. SG1, Sreetosh Goswami (SG2), DS designed the experiments. DS and RP performed all the chemical reactions and characterized the products, SG2 performed the Raman measurement, RB performed the DFT-calculations, AD synthesized the nanoparticles and characterized them. MG, VB conceptualized the siderophore analogy and performed relevant theoretical calculations. SG2, DS, RB, AP, SN, MG, VB, TV and SG1 analyzed experimental and theoretical data and wrote the paper. NP supervised the synthesis and characterization of Pt-NPs, SN supervised the theoretical calculations, and SG1 supervised the entire project.

## Conflicts of interest

There are no conflicts to declare.

## Supplementary Material

SC-011-D0SC02683B-s001

SC-011-D0SC02683B-s002
