# Supplementary material for: Size-selective Pt siderophores based on redox active azo-aromatic ligands
Source: Chem Sci. 2020 Aug 20;11(34):9226–36. doi: 10.1039/d0sc02683b (PMC8163438; doi:10.1039/d0sc02683b)
Supplement: SC-011-D0SC02683B-s001 [file SC-011-D0SC02683B-s001.pdf]

Electronic Supplementary Material (ESI) for Chemical Science.
This journal is © The Royal Society of Chemistry 2020

Electronic Supplementary Information

Size-selective Pt siderophores based on redox active azo-aromatic ligands

Debabrata Sengupta[a,†,#], Sreetosh Goswami[b,c,#,*], Rajdeep Banerjee[d,#], Matthew J. Guberman-Pfeffer[e], Abhijeet Patra[b], Anirban Dutta[f], Rajib Pramanick[a], Shobhana Narasimhan[d,*], Narayan Pradhan,[f,*], Victor Batista[e,g], T. Venkatesan[b,c,h,i,j], and Sreebrata Goswami[a,*].

[a]School of Chemical Sciences, Indian Association for the Cultivation of Science, Jadavpur, Kolkata 700032, India.

[b]NUSNNI-NanoCore, National University of Singapore, Singapore 117411, Singapore.

[c]NUS Graduate School for Integrative Sciences and Engineering (NGS), National University of Singapore, Singapore 117456, Singapore.

[d]Theoretical Sciences Unit and School of Advanced Materials, Jawaharlal Nehru Centre for Advanced Scientific Research, Jakkur, Bangalore 560064, India.

[e]Department of Chemistry, Yale University, 225 Prospect Street, New Haven, Connecticut 06520, United States

[f]School of Materials Sciences, Indian Association for the Cultivation of Science, Jadavpur, Kolkata 700032, India.

[g]Energy Sciences Institute, Yale University, 810 West Campus Drive, West Haven, Connecticut 06516, United States

[h]Department of Physics, National University of Singapore, Singapore 117542, Singapore.

[i]Department of Electrical and Computer Engineering, National University of Singapore, Singapore 117583, Singapore.

[j]Department of Materials Science and Engineering, National University of Singapore, Singapore 117575, Singapore.

†**Present address:** Department of Chemistry and Biochemistry, University of Texas at El Paso, El Paso, Texas 79968, United States.

Table of Content	*Page No*
Section S1- Size Distribution Analysis	S3-S7
Figure S1.	S3
Figure S2.	S4
Figure S3.	S5
Figure S4.	S5
Figure S5.	S6
FigureS6.	S6
Figure S7.	S7
FigureS8.	S7
Section S2- In-situ ***Spectroscopic Characterizations***	S8-S12
Figure S9	S8
Figure S10	S9
Figure S11	S10
Figure S12	S10
Figure S13	S11
Figure S14	S12
Section S3- Characterization of the Isolated Complex	S13-S17
X-Ray Crystallography	S13
Table S1	S14
Table S2	S15
Figure S15	S16
Section S4- Computational Details	S17-S21
Determination of maximum cluster size (n_{max}) for etching	S17
Modification of etching-size threshold with different ligands	S18
Figure S16	S18
Table S3	S19
Table S4	S19
Equation used for fitting	S19
Figure S17	S20
Figure S18	S21
Section S5- Control experiments	S22-S26
Figure S19	S22
Figure S20	S23
Figure S21	S24
Figure S22	S25
Figure S23	S26
Reference	S26

Section-S1: Size Distribution Analysis

The NP sizes were analyzed in MATLAB via image process. A representative is shown in Figure S1. From the measured TEM image the edges are detected using Canny edge detection algorithm. Individual structures thus identified were fitted with circles. The fitting efficiency, given by the ratio of the sum of the area under the fitted circles to the sum of the area under the structures was >94%. The diameter of these fitted circles was assumed to represent the NP size.

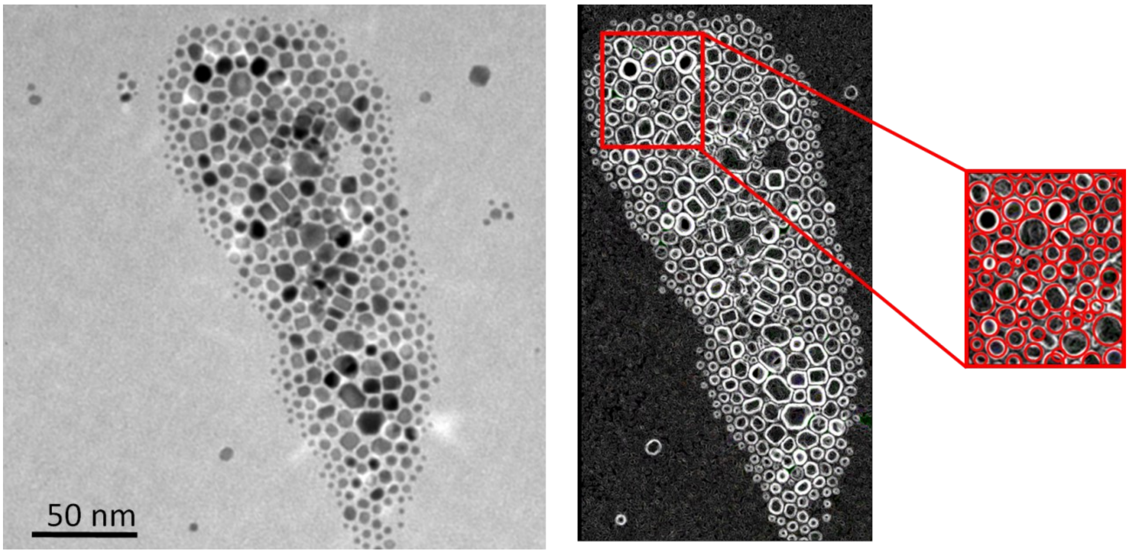

Figure S1. Determination of NP size- a. Measured TEM, b. Detected edged c. Size estimation.

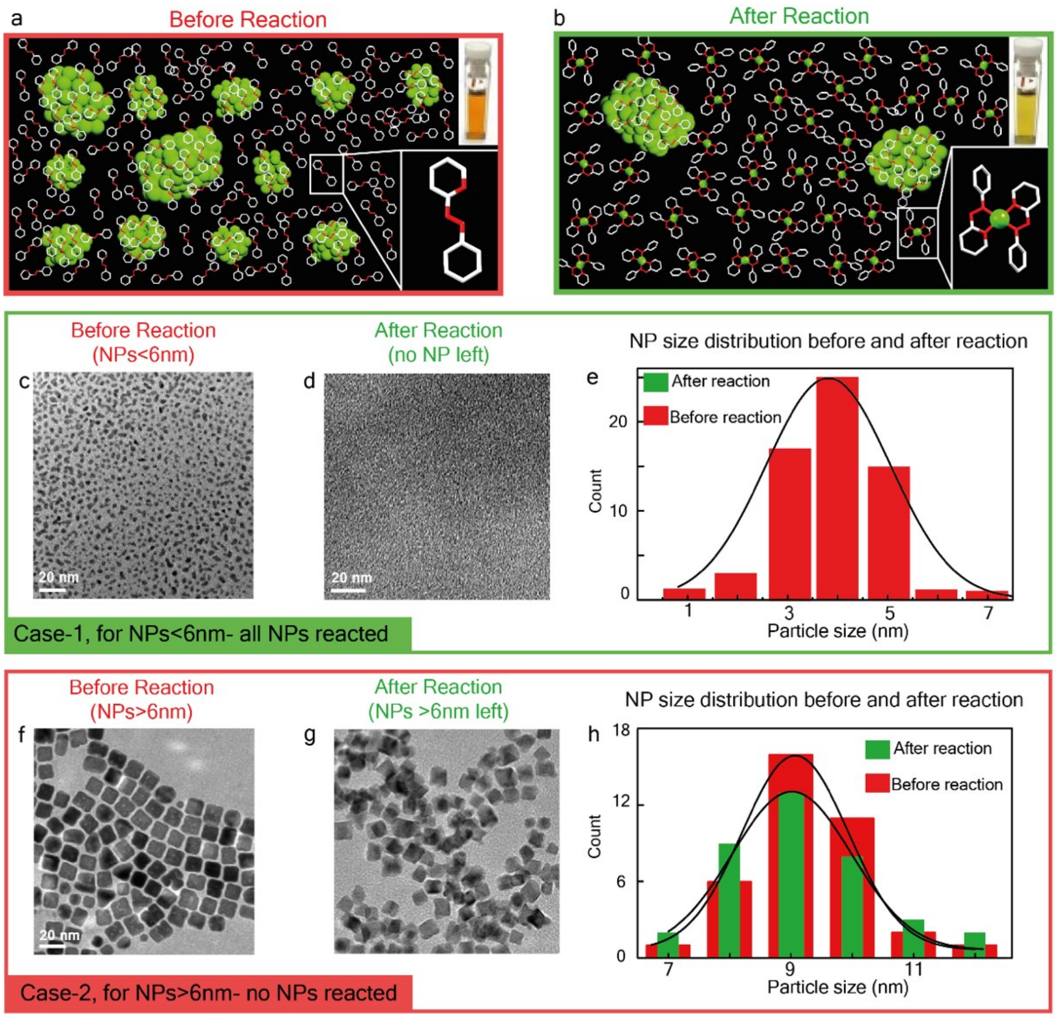

Figure S2. Size dependent chemical reactivity of Pt Nanoparticles – (a,b) Schematic presentation of chemical reactivity of Pt NPs with L_1: smaller size NPs (d ≤ 6 nm) react but larger NPs do not. The reaction causes a change in color from red to green. (c,d) TEM images before and after reaction between NPs with d ≤ 6 nm and L_1 and (e) the corresponding NP size distribution in pre- and post-reaction samples. (f-h) Same as (c-e) for NPs with d > 6 nm.

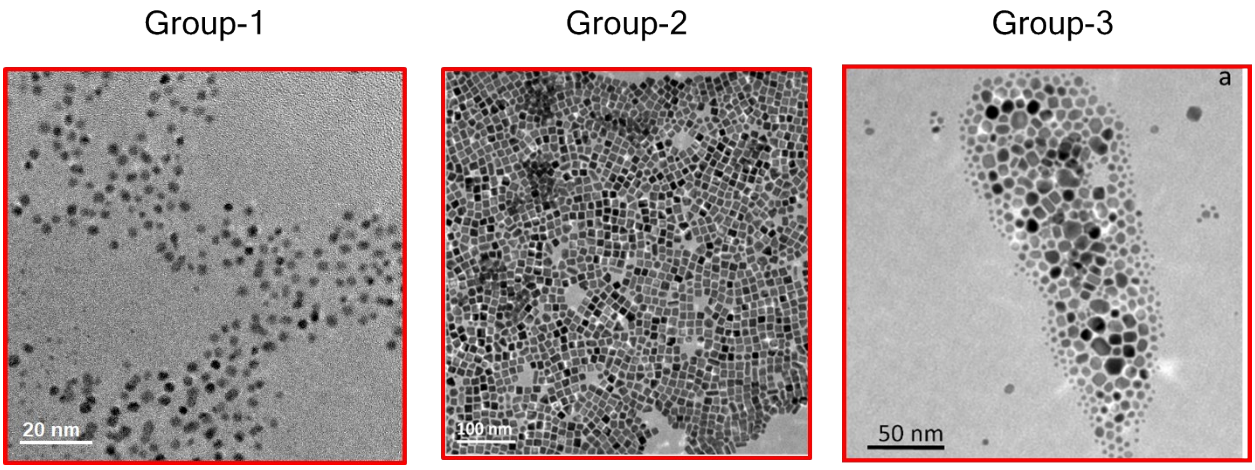

Figure S3: NP Size variation – Nano-particles of different size range (group- 1, 2 and 3) and different morphologies. Note- the scalebars are different (chosen to shaow the maximum area with reasonable spatial resolution).

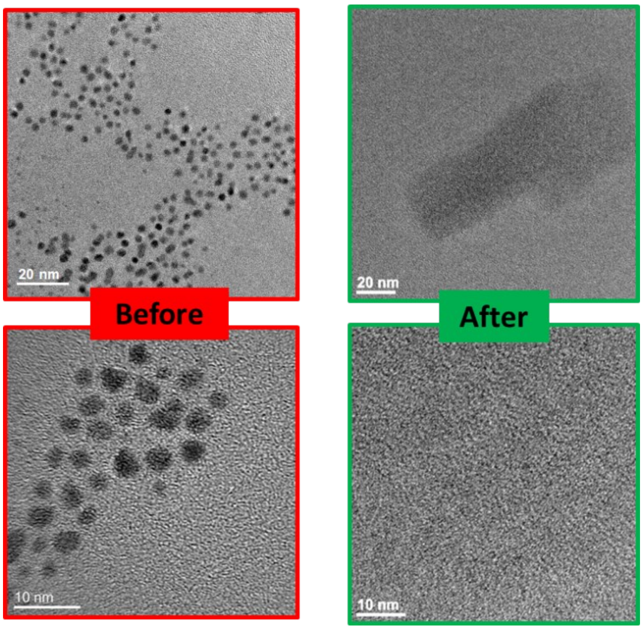

Figure S4. TEM measurements. TEM images of before and after reaction of **Group 1** nanoparticles in different sets.

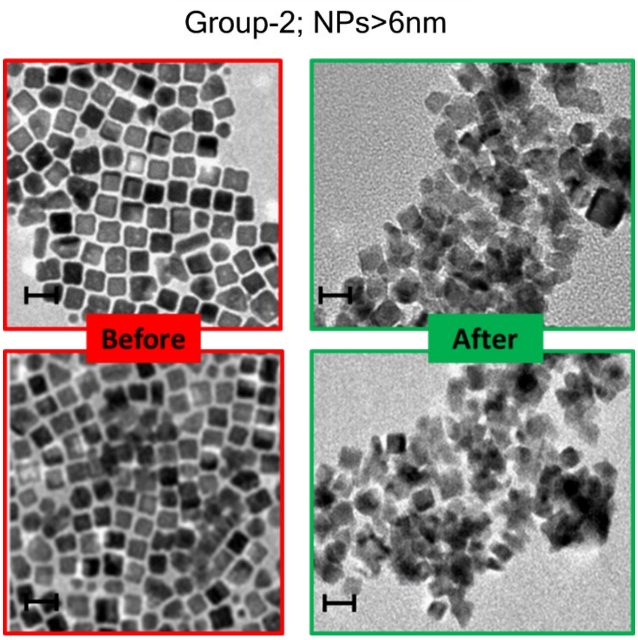

Figure S5. **TEM measurements**. TEM images of before and after reaction of **Group 2** nanoparticles in different resolution.

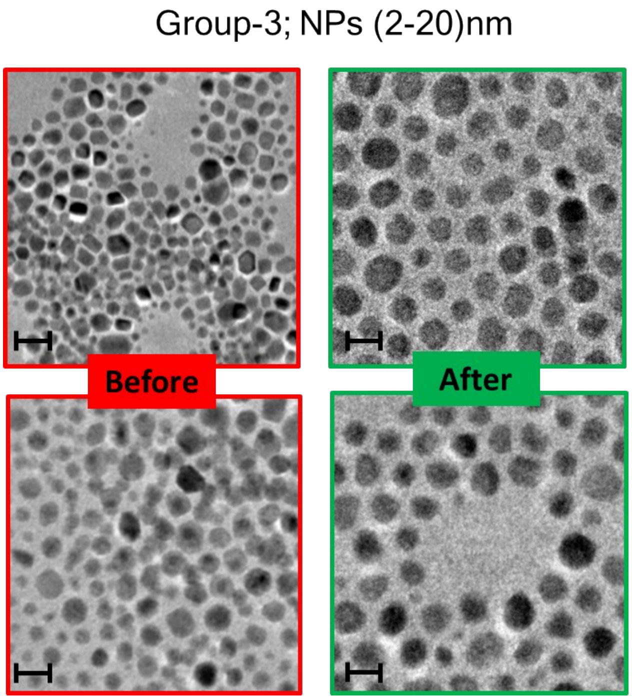

Figure S6. **TEM measurements**. TEM images of before and after reaction of **Group 3** nanoparticles in different resolution.

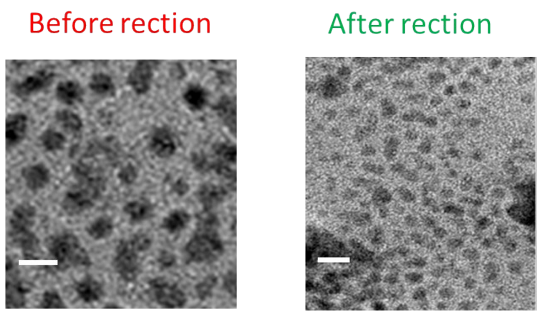

Figure S7. Incomplete etching. TEM images before reaction and after incomplete etching showing reduction in NP size. The scalebar is 5nm.

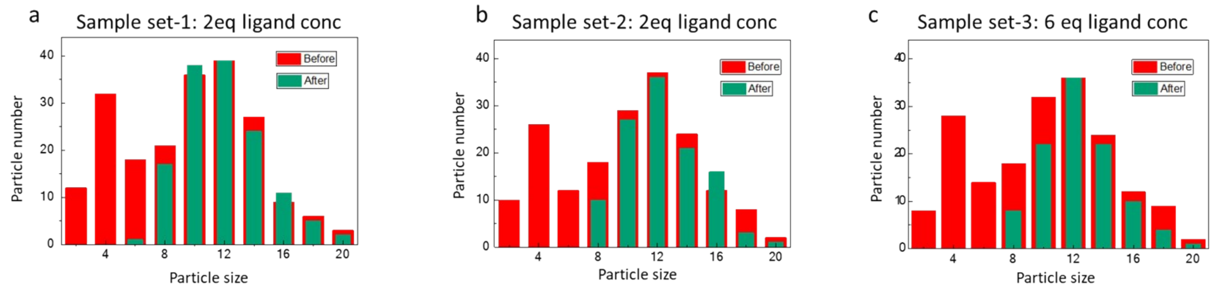

Figure S8. Sample and concentration dependence of switching threshold. Estimation of size threshold reactivity using NP samples of size ranging from 2-20nm. (a, b) We show two different sample sets with same ligand concentration (2 equivalent) and observation of identical size threshold. (c) Besides taking a different sample set, the concentration was increased by a factor of 3 (*i.e.* 6 equivalent) to verify an unchanged size threshold.

Section S2 *In-situ* spectroscopic characterizations

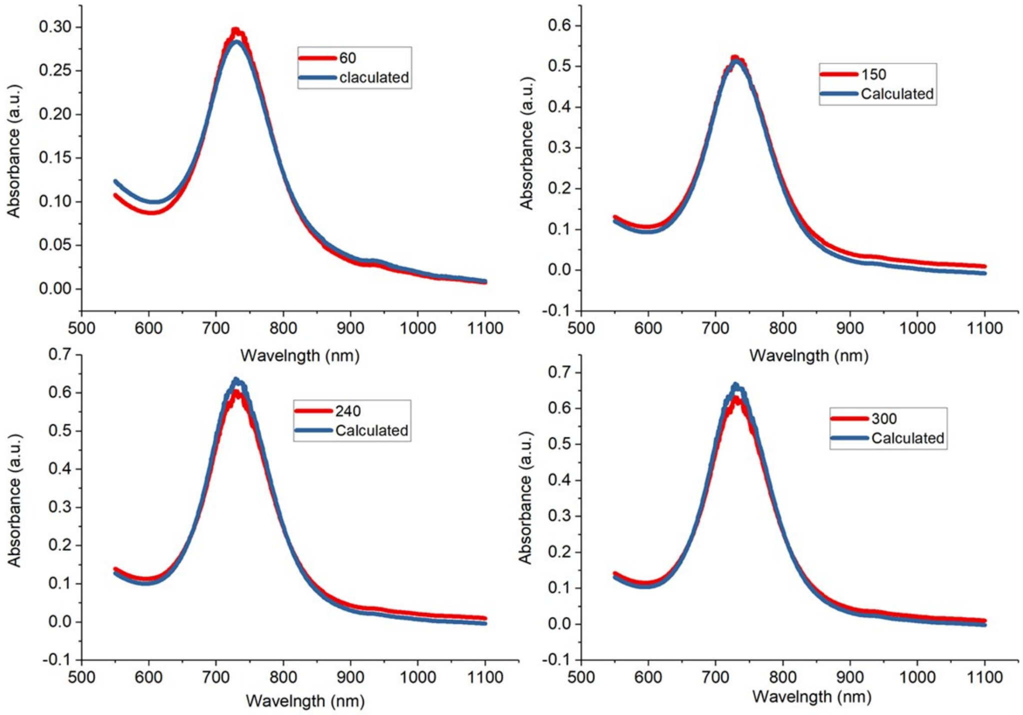

Figure S9. ***In-situ* Vis-NIR spectra of $Pt(L_1^{\bullet-})_2$ as a linear combination of the initial and final spectra**. The spectra measured in between 30 and 300 minutes can be expressed as a linear combination of the initial and final spectra. Here the calculated spectra are derived using $\frac{ai \times (initial\,spectrum) + af \times (final\,spectrum)}{ai + af}$ where ai and af are proportionality constants.

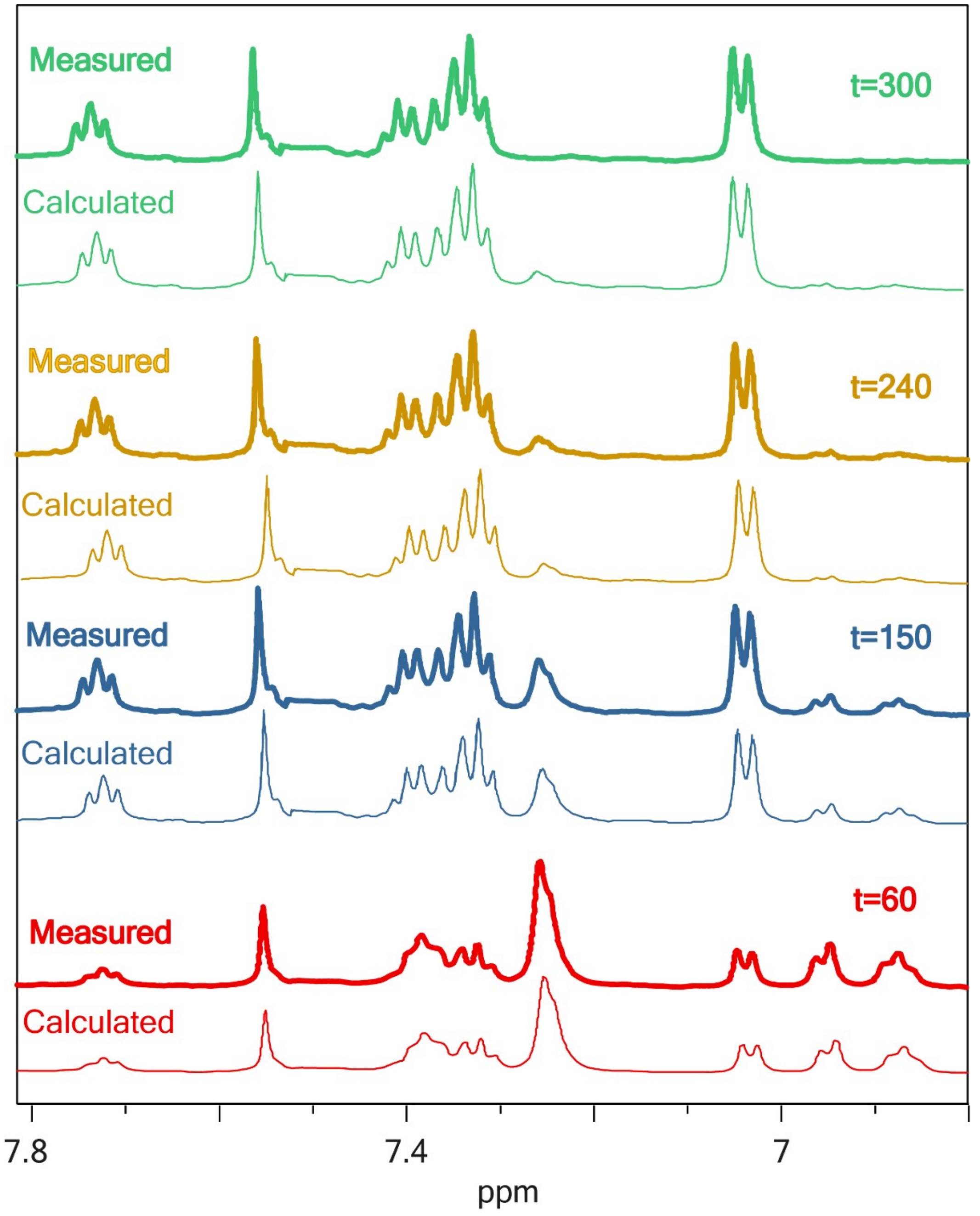

Figure S10. ***In-situ* NMR spectra as a linear combination of the initial (of L_1) and final (of $Pt(L_1^{\bullet -})_2$) spectra**. The spectra measured in between 30 and 300 minutes can be expressed as a linear combination of the measured initial and final spectra showing a near perfect match. Here the calculated spectra are derived using $\frac{ai \times (initial\ spectrum) + af \times (final\ spectrum)}{ai + af}$ where ai and af are proportionality constants.

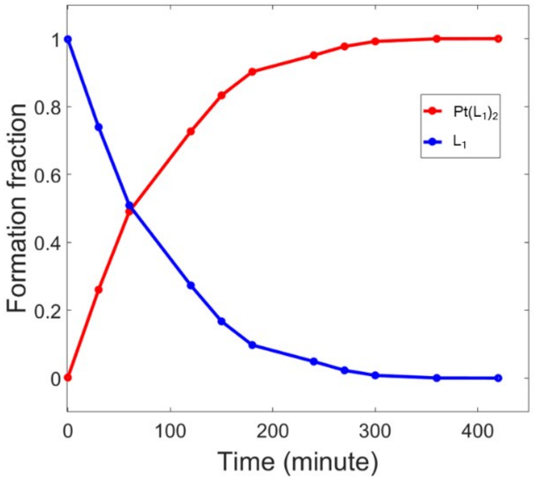

Figure S11. **Reaction rate estimation**. From the ai and af estimated from Figures S9 and S10, we computed the percentage of L_1 and $Pt(L_1)_2$ in the solution with Pt NPs (≤ 6nm) at different points of time.

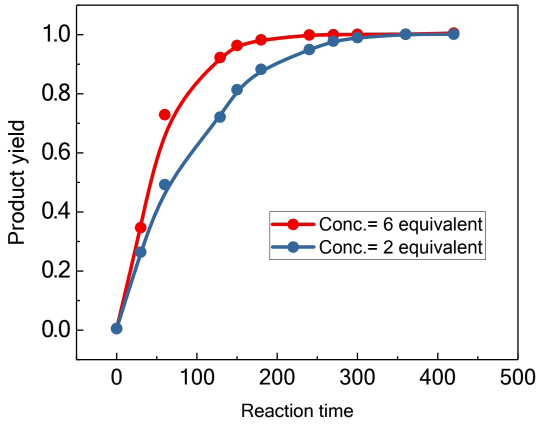

Figure S12. Reaction rate dependence on concentration. The rate of reaction of NPs< 6nm for different ligand concentration.

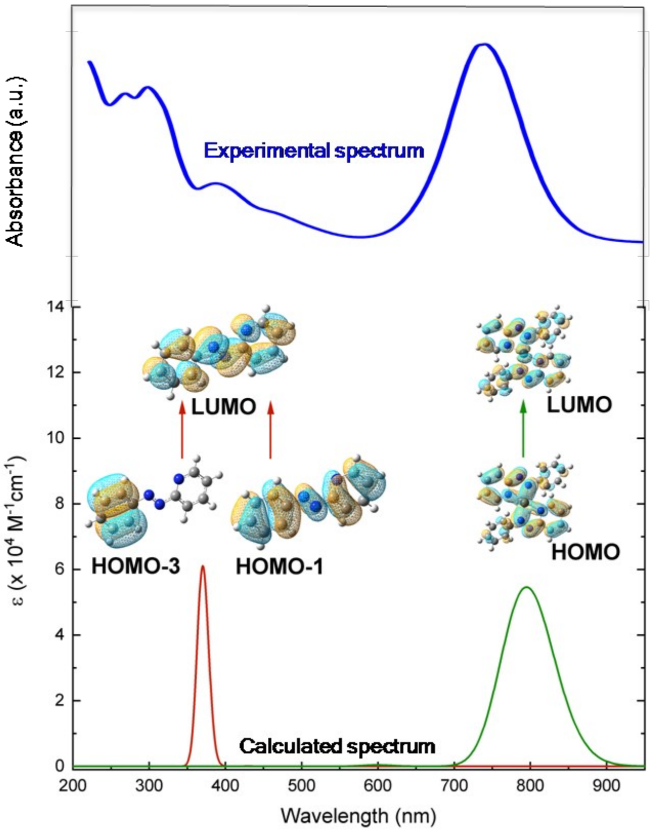

Figure S13. **Correspondence between measured and calculated UV-vis**. The experimental spectrum of the isolated $Pt(L_1^{\bullet-})_2$showing peaks at 315 and 725 nm consistent with the two transition peaks calculated at 340 and 790 nm.

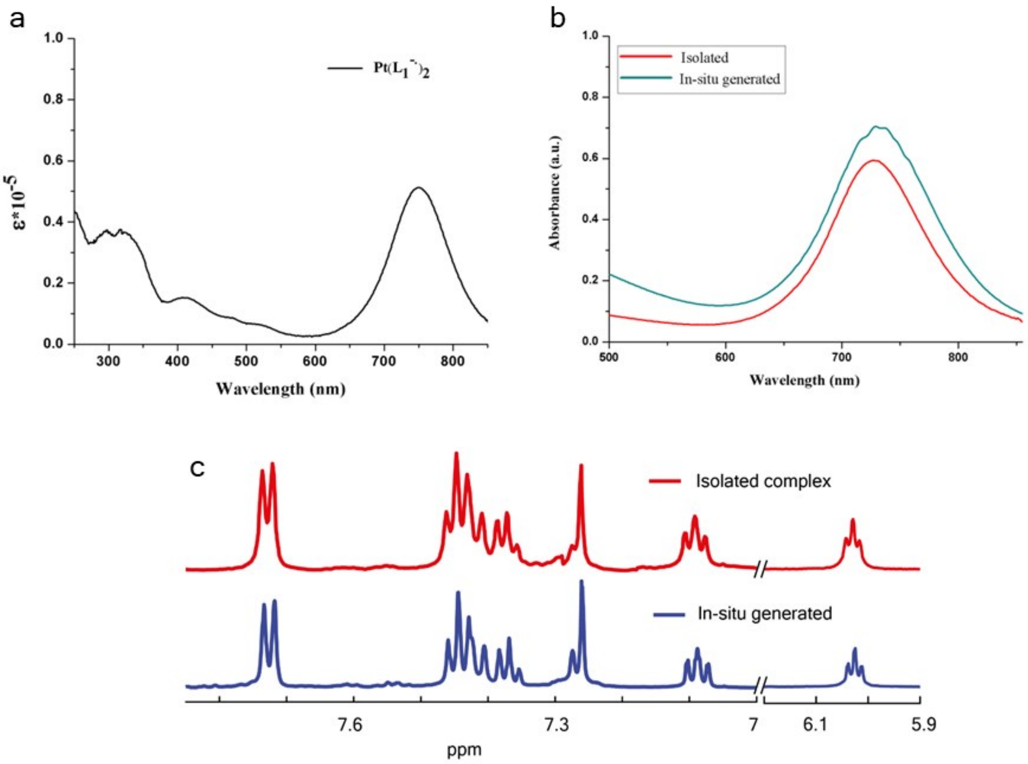

Figure S14. Comparison of UV-vis spectra.(a) UV-vis spectrum of 10^{-5} M Pt($L_1^{•-}$)$_2$ in dichloromethane(b) Comparison between isolated Pt($L_3^{•-}$)$_2$ and *in-situ* generated Pt($L_3^{•-}$)$_2$ measured under identical conditions; using similar solution composition (methanol+PEG). (c) Comparison between the ^{1}H NMR spectra (in $CDCl_3$) of the isolated Pt^{II}($L_1^{•-}$)$_2$ and in-situ generated species shown in Figure 3a (at t > 350 min).

Section S3 Characterization of the isolated complex

X-ray Crystallography

Single crystal X-Ray crystallographic data for $Pt^{II}(L_1^{\bullet-})_2$ was collected in Table S1. Suitable X-ray quality single crystals of the complex $Pt^{II}(L_1^{\bullet-})_2$ was obtained by slow diffusion of a dichloromethane solution of the complex into hexane. A Bruker SMART APEX-II diffractometer equipped with graphite-monochromated Mo Kα radiation (λ = 0.71073 Å) was used for X-ray data collection. The collected data were corrected for Lorentz polarization effects. A total of 12971 reflections were collected, of which 2216 were unique (R_{int} = 0.041), satisfying $I > 2\sigma(I)$ criterion, and were used in subsequent analysis. The structure was solved by employing the SHELXS-2013 program[1]package and was refined by full-matrix least squares based on F^2 (SHELXL-2013).

All hydrogen atoms were added in calculated positions.

Table S1. Single crystal X-Ray crystallographic data table of $Pt(L_1^{\bullet-})_2$ complex.

	$Pt^{II}(L_1^{\bullet-})_2$
CCDC NO.	1042504
Empirical formula	$C_{22}H_{18}N_6Pt$
molecular mass	561.50
temperature (K)	293
crystal system	Monoclinic
space group	*P*21/*c*
a (Å)	9.8643(19)
b (Å)	9.9442(19)
c (Å)	10.655(2)
α(deg)	90
β (deg)	112.053(4)
γ (deg)	90
V (Å^3)	968.7(3)
Z	2
D_{calcd}(g/cm^3)	1.925
cryst. dimens. (mm)	0.14x0.16x0.18
θ range for data coll. (deg)	2.2 –27.5
GOF	0.86
reflns. Collected	12971
Uniq.reflns.	2216
final*R*indices [$I > 2\sigma(I)$]	R = 0.0229<br>wR2 = 0.0692

Table S2. Optimized Parameters. Experimental and optimized bond distances (Å) and bond angles (deg) of the complex, $Pt(L_1^{\bullet-})_2$.

**Bond Parameters**	$Pt^{II}(L_1^{\bullet-})_2$	
	**Experimental**	**Theoretical**
Pt1-N1	2.006(3)	2.0676
Pt1-N3	1.965(3)	2.0650
Pt1-N1a	2.006(3)	2.0677
Pt1-N3a	1.965(3)	2.0650
N2-N3	1.335(5)	1.3284
N1- Pt1-N3	76.59(12)	76.4642
N1a-Pt1-N3	103.41(12)	104.6774

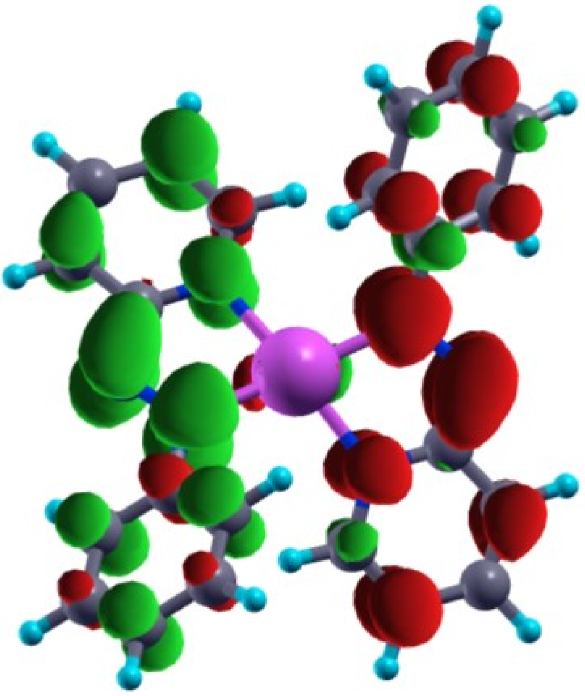

Figure S15. Spin density plot of the complex, $Pt^{II}(L_1^{\bullet-})_2$.

Section S4 Computational details

Determination of maximum cluster size (n_{max}) for etching

The sublimation energy $E_{\mathrm{sub}}(n)$is defined as the energy required to remove a Pt atom from a Pt_n cluster. It is defined as:

$$E_{\mathrm{sub}}(n) = -[E(\mathrm{Pt}_n) - \mathrm{E}(\mathrm{Pt}_{n\text{-}1}) - E(\mathrm{Pt})] \qquad (1)$$

Where $E(X)$ is the total energy of the system X; here, Pt_n is a gas-phase cluster containing n Pt atoms.

The binding energy of the $Pt^{II}(L_1^{\bullet-})_2$ complex is given by:

$$E_b(\mathrm{Pt}^{\mathrm{II}}(\mathrm{L}_1^{\bullet-})_2) = -[E(\mathrm{Pt}^{\mathrm{II}}(\mathrm{L}_1^{\bullet-})_2) - E(\mathrm{Pt}) - 2E(\mathrm{L}_1)] \qquad (2)$$

A positive/negative slope of the graph of ΔE vs. n (see Figure 5 of the main text) indicates that etching/sintering is favored. In the absence of the ligands, the slope of the graph is given by:

$$E(\mathrm{Pt}_n) - \mathrm{E}(\mathrm{Pt}_{n\text{-}1}) - E(\mathrm{Pt}) = -E_{\mathrm{sub}}(n) \qquad (3)$$

$E_{\mathrm{sub}}(n) > 0$, for all n, and indeed from Figure 5, we see that the slope of ΔE vs. n is always negative in the gas-phase, i.e., sintering is always favored in the gas-phase.

In the presence of the ligand L_1, the slope of ΔE vs. n can be written as:

$$E(\mathrm{Pt}_n) - \mathrm{E}(\mathrm{Pt}_{n\text{-}1}) - E(\mathrm{Pt}^{\mathrm{II}}(\mathrm{L}_1^{\bullet-})_2 + 2E(\mathrm{L}_1)$$
$$= - E_{\mathrm{sub}}(n) + E_b(\mathrm{Pt}^{\mathrm{II}}(\mathrm{L}_1^{\bullet-})_2) \ < 0 \text{ for } n<n_{\mathrm{max}}, \qquad (4)$$

Where n_{max} is the size of cluster at which the slope of ΔE vs. n becomes 0. In other words, the etching stops when $E_{\mathrm{sub}}(n_{\mathrm{max}}) = E_b(\mathrm{Pt}^{\mathrm{II}}(\mathrm{L}_1^{\bullet-})_2)$. This is the condition for the maximum cluster size n_{max} up to which etching takes place in the presence of the ligand L_1.

Modification of etching-size threshold with different ligands

We considered two classes of closely related ligands (Figure S13) : (i) a family of 2-(arylazo) pyridines (L); *viz.* 2-(phenylazo) pyridine (L_1), 2-(4-chlorophenylazo) pyridine (L_2), 2-(4-methylphenylazo) pyridine (L_3) and (ii) common di-imineligands; *viz.* 9,10-phenanthroline (Λ_1), 2-(phenylimino) pyridine (Λ_2) and 2,2'-bipyridine (Λ_3). We find, from DFT, that $E_b(Pt^{II}(L^{\bullet-})_2)$ for the ligands L_1-L_3 has values of 7.72, 7.66 and 8.29 eV, respectively whereas the E_b values for the Λ family of ligands ($E_b(Pt^{II}(\Lambda^{\bullet-})_2)$ with Λ_1-Λ_3) are smaller, *viz*., 5.68, 6.88 and 6.27 eV, respectively. Consequently, while the n_{max} values for the $L_{1\text{-}3}$ are 9930 (6.58 nm), 8511 (6.25 nm) and 8879 (6.34 nm) those for $\Lambda_{1\text{-}3}$ come out as 43 (1.07 nm), 336 (2.13 nm) and 193 (1.77 nm), respectively (see Table S3, S4). The n_{max} values for the L-series implies a threshold size of ~6 nm for this reaction while that for Λ-series come out as ~ 1 nm. Experimentally, all the ligands of L-series dissolve NPs ≤ 6 nm, while those of Λ-series do not. Since> 95% of the isolated NP have d ≥ 2 nm, the threshold for the Λ-series could not be experimentally detected. Nonetheless, this insight could guide further designing of ligands with different E_b values that can manipulate the NP size-threshold of the reaction.

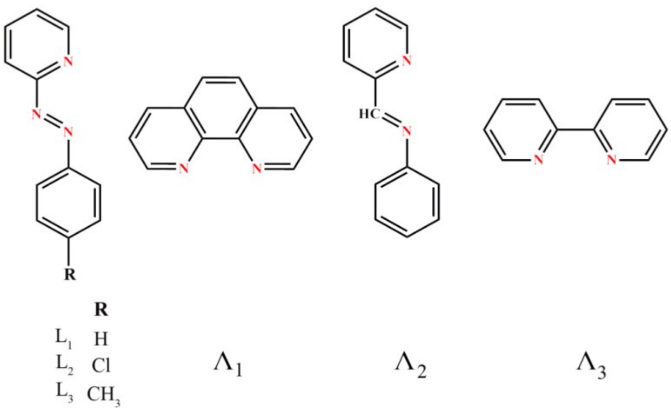

Figure S16. Schematic representation of the used organic ligands.

Table S3. Maximum cluster size for etching and binding energies. Comparison of the maximum cluster size n_{max} in Pt_n, where etching stops, cluster sizes (in nm) corresponding to n_{max} and the binding energies of the etched Pt atom to the corresponding ligands

Ligands	n_{max}	Cluster size (nm)	E_b (eV)
L_1	9,930	6.58	7.72
L_2	8,511	6.25	7.66
L_3	8,879	6.34	7.68
Λ_1	43	1.07	5.68
Λ_2	336	2.13	6.88
Λ_3	193	1.77	6.27

Table S4. **HOMO-LUMO calculation by DFT.** Comparison of highest and lowest occupied molecular orbitals of selected π-acceptor ligands

Ligand	HOMO (eV)	LUMO(eV)	E_g(eV)
L_1	-6.655	-2.403	4.252
L_2	-6.393	-2.802	3.591
L_3	-6.83	-2.393	4.437
Λ_1	-6.708	-3.301	3.407
Λ_2	-6.786	-3.453	3.333
Λ_3	-6.568	-3.203	3.365

Equation used for fitting

To determine ΔE vs. n, we could perform DFT calculations only up to n = 2057, beyond which results are obtained by a quadratic extrapolation determined by the following general formula:

$$\Delta E = a_0 n^2 + a_1 n + a_2 \quad (5)$$

where a_0, a_1 and a_2 are numerical fitting parameters. ΔE ist he total energy and n is the nanoparticle size. In general, such a quadratic curve gives a very good fit to calculated values of ΔE vs. n, especially for higher n values. We verified the efficiency of our fitting for Λ_1, Λ_3 ligand systems (Figure S14) where $n_{th} < 200$ that allow us to quantum mechanically compute the ΔE values for a larger number of n around n_{th} justifying there liability and efficiency of our fitting protocol. Thereafter, using the same equation, we extrapolated the curves for higher values on n for L-ligand system where n_{th}>8000 that are beyond our scope of calculation.

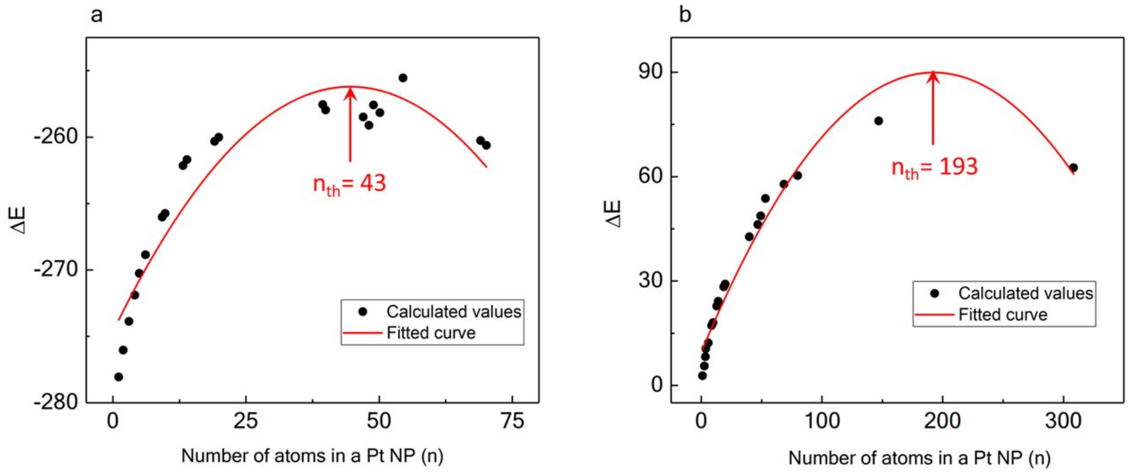

Figure S17. Δ*E* vs. *n* plots (Λ_1 and Λ_3). Δ*E* vs. *n* plots in the presence of (a) Λ_1 and (b) Λ_3 ligand. The corresponding n_{max} values found by fitting are shown in red. At lower values on *n*, *ΔE* is expected to be sensitive to the geometry and shape and deviations from smooth trends is quite normal. As shown in Figure S12, *ΔE(n)* curve for Λ_1 and Λ_3 can be bestfitted with quadratic polynomial justifying the use of the fitting equation 5. For the fitting presented in Figure 5f, the values of a_0, a_1 and a_2 are -359.477, 1.78944 and -9.01024e^{-5} that provide a R^2 = 0.999824 till n = 2057 which is a near ideal fit and corresponds well to the experimentally observed value.

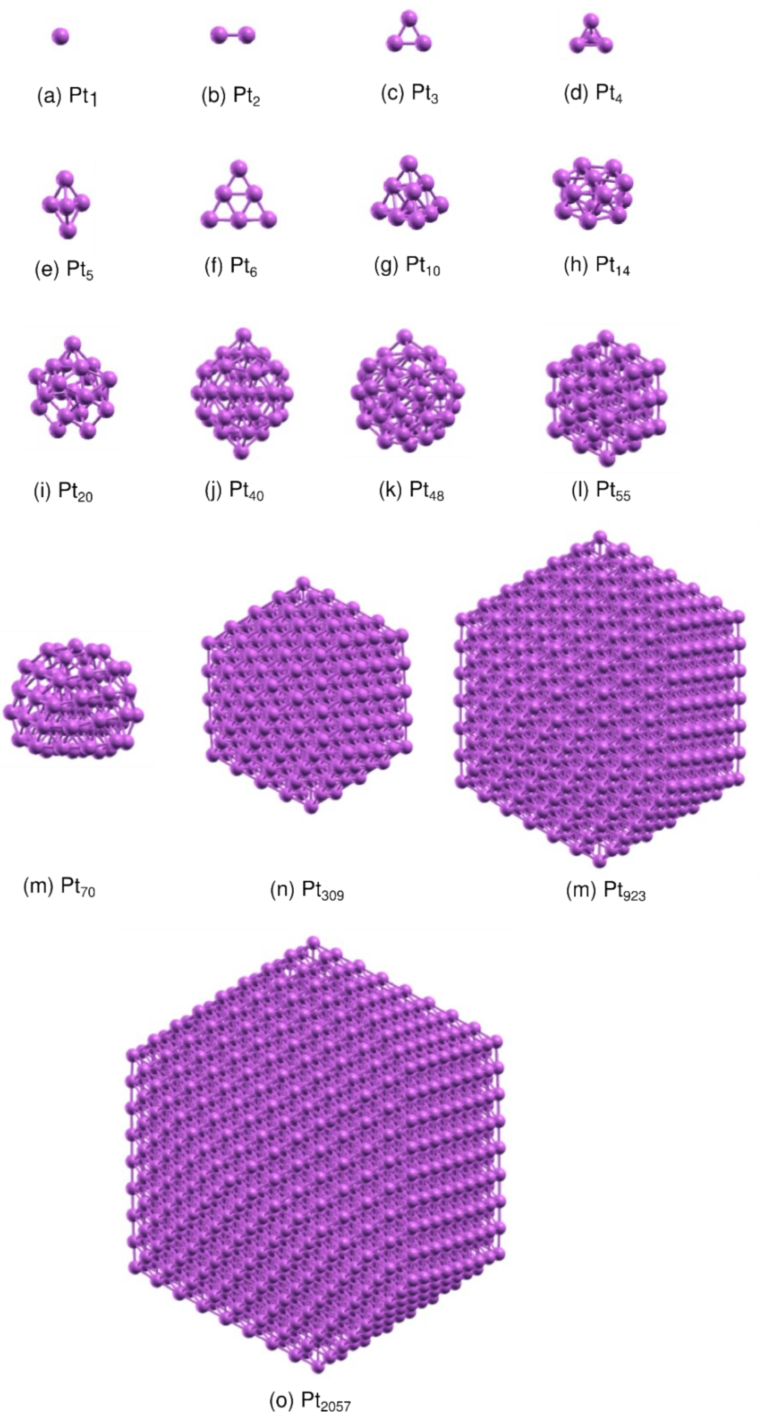

Figure S18. Structure of the nanoparticles. The optimized structures of the Pt nanoparticles

Section S5 Control experiments

(a)

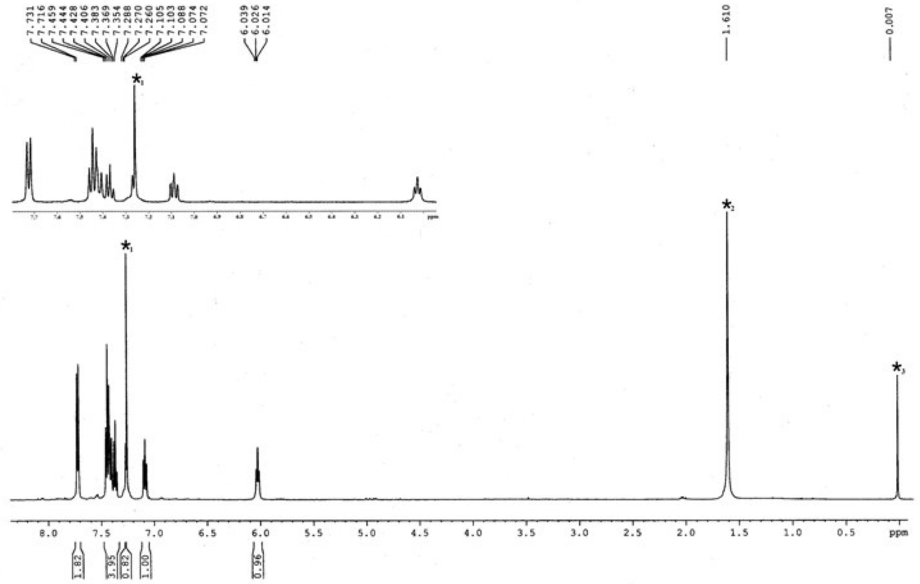

(b)

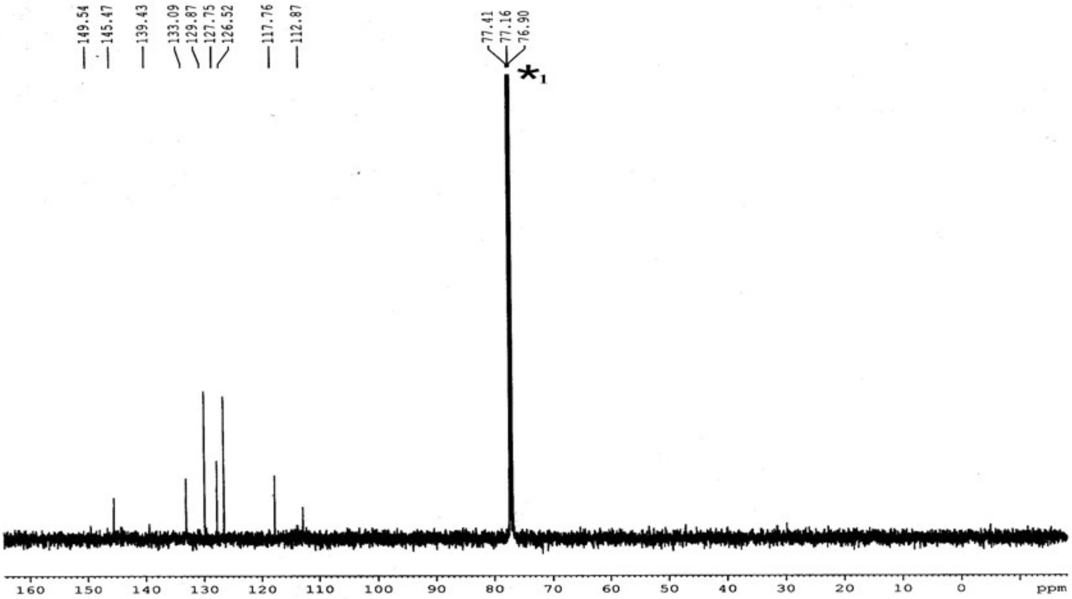

Figure S19.^{1}H NMR analysis of the isolated complex, of $Pt(L_1^{\bullet-})_2$. (a) ^{1}H NMR spectrum of $Pt^{II}(L_1^{\bullet-})_2$ in $CDCl_3$ solution. (Inset: Expansion of aromatic region, $*_1$= solvent, $*_2$= water, $*_3$= TMS), (b) ^{13}C NMR spectrum of $Pt^{II}(L^{\bullet-})_2$ in $CDCl_3$ solution ($*_1$ = solvent).

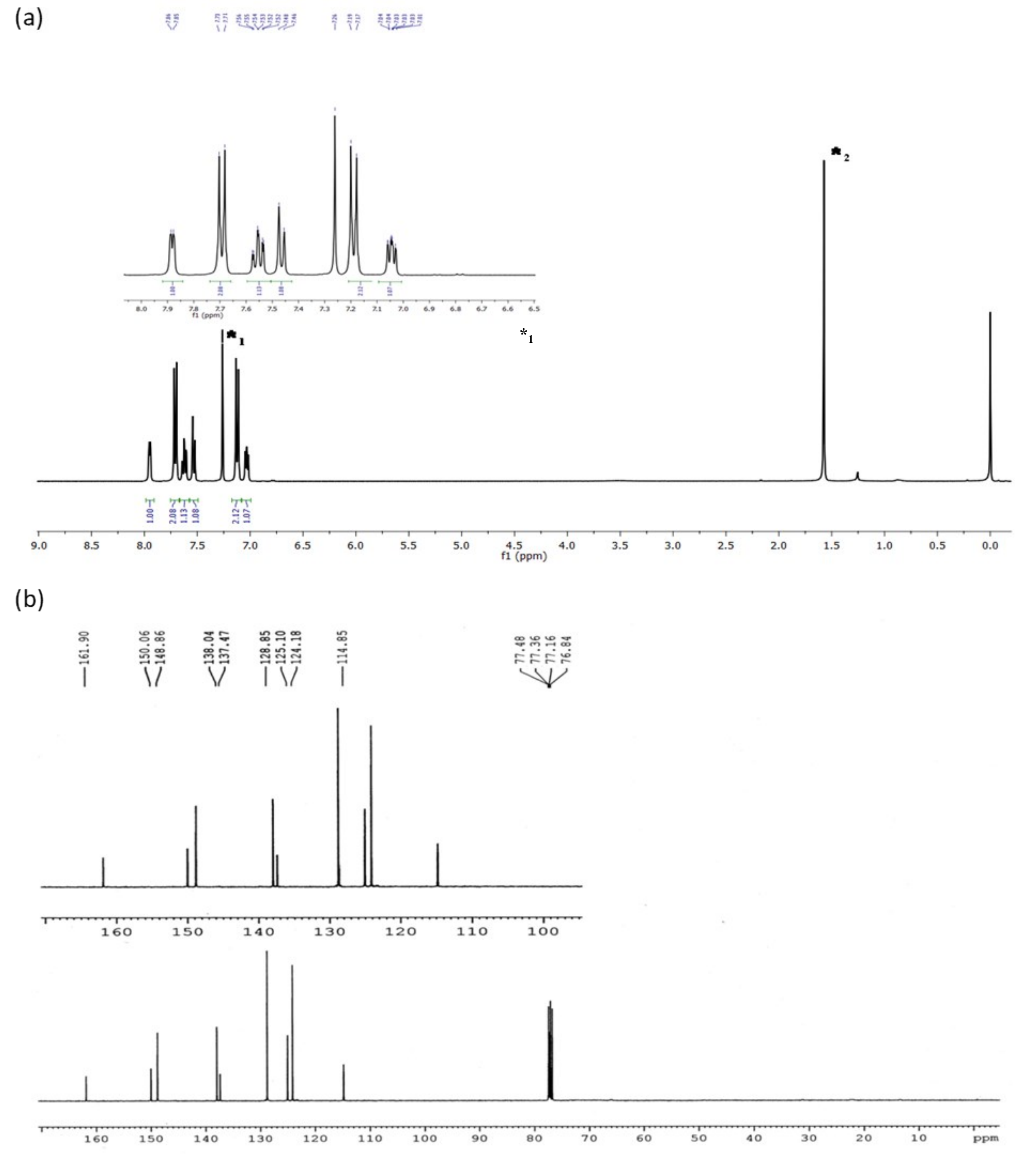

Figure S20.^{1}H NMR analysis of the isolated complex, of Pt($L_2^{\bullet -}$)$_2$. (a)^{1}H NMR spectrum of Pt($L_2^{\bullet -}$)$_2$in $CDCl_3$ solution (Inset: Expansion of aromatic region); (b)^{13}C NMR spectrum of Pt($L_2^{\bullet -}$)$_2$in $CDCl_3$ solution (Inset: Expansion of aromatic region) =solvent residue $*_2$=water.

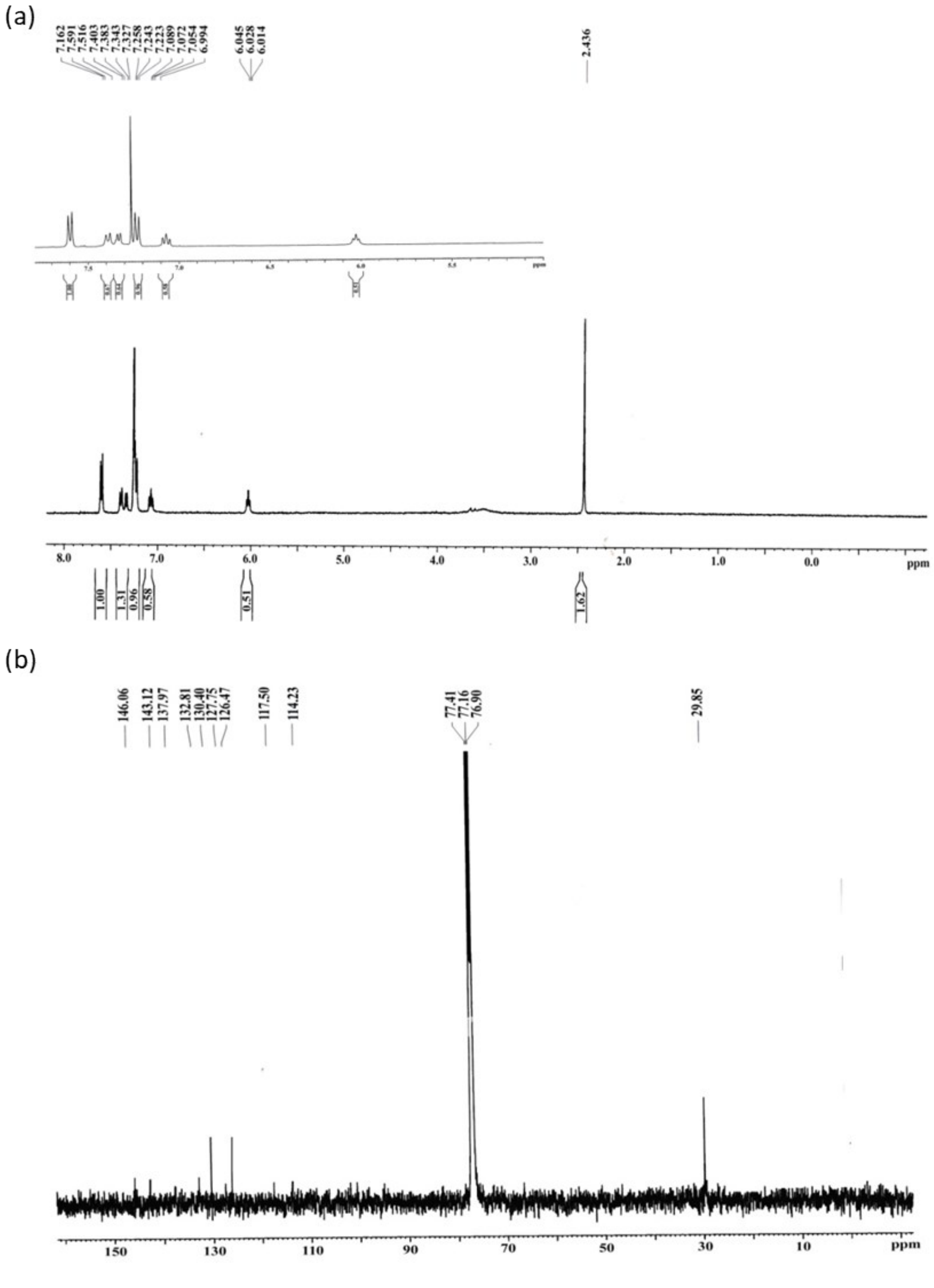

Figure S21.^{1}H NMR analysis of the isolated complex, $Pt(L_3^{\bullet -})_2$. (a)^{1}H NMR spectrum of $Pt(L_3^{\bullet -})_2$ in $CDCl_3$ solution (Inset: Expansion of aromatic region); (b)^{13}C NMR spectrum of $Pt(L_3^{\bullet -})_2$in $CDCl_3$ solution (Inset: Expansion of aromatic region) ($*_1$ = solvent residue).

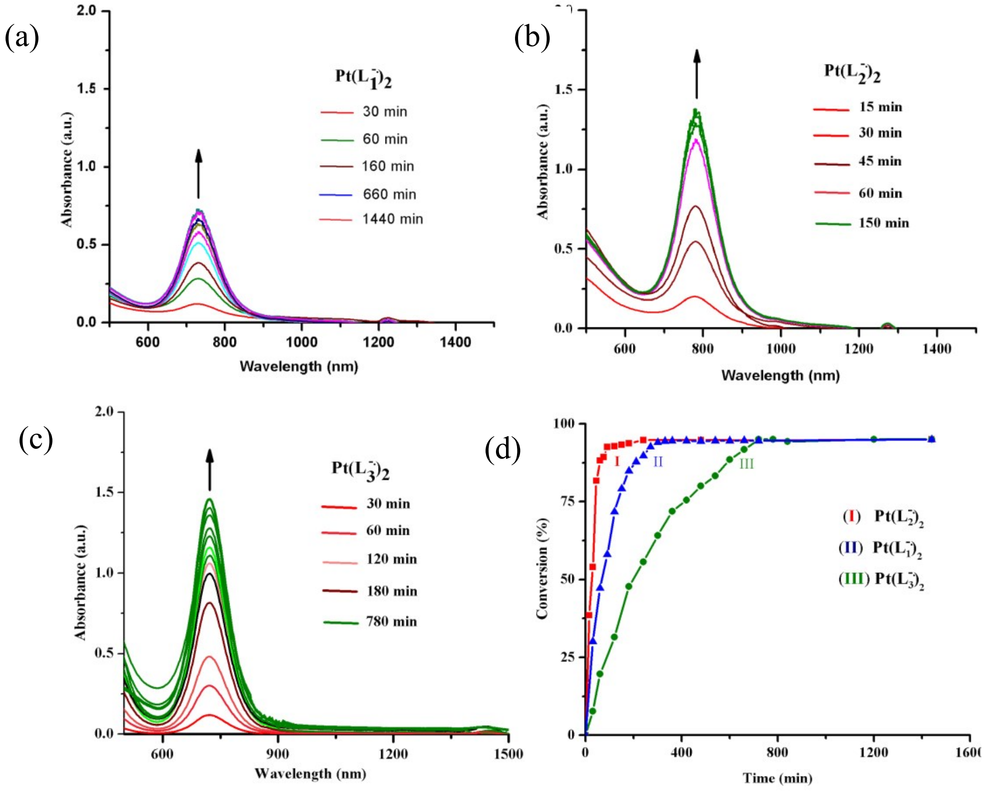

Figure S22. Time wise successive absorption spectra. Reaction of different ligands with Group 1 nanoparticles: (a) formation of $Pt(L_1^{\bullet-})_2$(b) formation of $Pt(L_2^{\bullet-})_2$ (c) formation of $Pt(L_3^{\bullet-})_2$ (d) Percentage of conversion for the formation of the three complexes with time using different ligands.

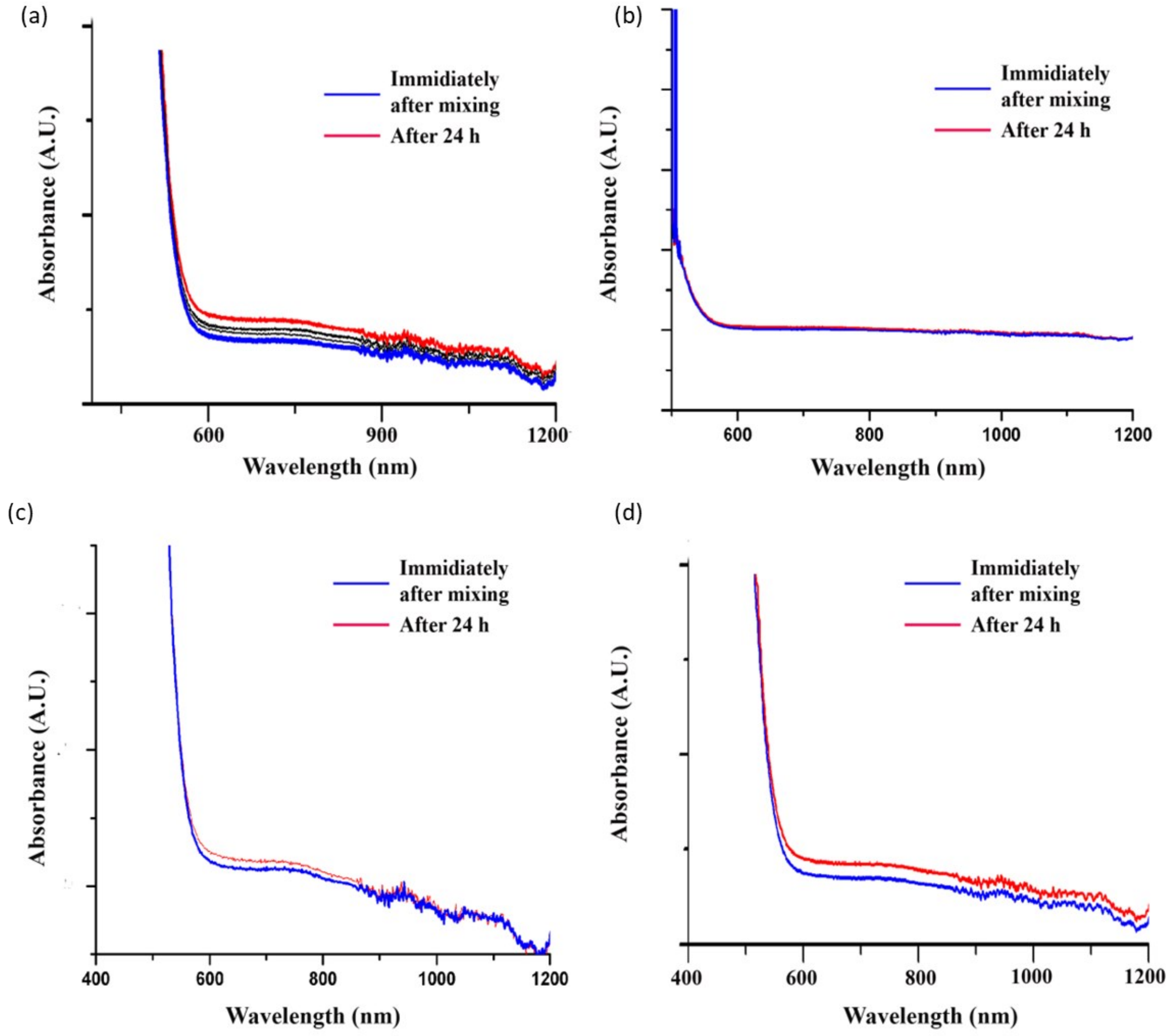

Figure S23. Use of absorption spectra as marker for different mixtures of reactants: (a) Reaction between **Group 2** Pt-NPs and L_1 (b) Reaction between **Group 2** Pt-NPs and 9,10-phenanthroline (Λ_1), (c) Reaction between **Group 2** Pt-NPs and 2,2'-bipyridine(Λ_3), (d) Reaction between **Group 2** Pt-NPs and 2-(phenylimino) pyridine (Λ_2)**.**

There is virtually no change of spectrum even after 24 h. This clearly indicates that none of the above mixtures resulted in formation of any product. This is as expected as discussed in the main text.

References

1. G. M. Sheldrick, *Acta Crystallogr., Sect. A: Found. Crystallogr.*, 2008, **64**, 112
